# Lactylation-driven metabolic reprogramming promotes osteosarcoma malignancy via HDGF-mediated proliferation and immune modulation

**DOI:** 10.3389/fimmu.2026.1836254

**Published:** 2026-05-20

**Authors:** Shidong Hu, Ling Wu, Jianhua Wang, Fengsheng Dai

**Affiliations:** 1 Department of Spine Surgery, Zhongda Hospital Southeast University, Nanjing, China; 2 Department of Orthopedic Surgery, The Second Affiliated Hospital of Chongqing Medical University, Chongqing, China; 3 Department of Oncology, The First Affiliated Hospital of Dali University, Dali, China; 4 Chongqing Key Laboratory for the Mechanism and Intervention of Cancer Metastasis, Chongqing University Cancer Hospital, Chongqing University, Chongqing, China; 5 Department of Hepatobiliary Pancreatic Tumor Center, Chongqing University Cancer Hospital, Chongqing, China

**Keywords:** hdgf, immune cell, lactylation, osteosarcoma, single-cell RNA sequencing, tumor heterogeneity

## Abstract

**Background:**

Osteosarcoma (OS) is characterized by significant intratumoral heterogeneity and a remodeled immune microenvironment. However, the mechanisms through which metabolic reprogramming drives tumor progression are not fully understood. Lactylation, an epigenetic modification derived from lactate, has recently emerged as a key regulator of tumor metabolism and immune modulation.

**Methods:**

We employed an integrative multi-omics approach, combining single-cell RNA sequencing, spatial transcriptomics, and machine learning-based prognostic modeling to decipher lactylation-associated networks in OS. Key drivers were identified through co-expression network analysis and SHapley Additive exPlanations (SHAP) interpretability frameworks, followed by experimental functional validation and virtual screening for potential therapeutics.

**Results:**

We identified a distinct tumor subpopulation exhibiting high lactylation activity, genomic instability, and stem-like properties, which is associated with unfavorable patient prognosis. Network and SHAP analyses pinpointed hepatoma-derived growth factor (HDGF) as a central regulator within this axis. Functional studies demonstrated that HDGF knockdown potently inhibited OS cell proliferation, migration, invasion, and tumor growth *in vivo*. Spatial transcriptomics confirmed the colocalization of elevated lactylation activity and HDGF expression within tumor regions, linking metabolic reprogramming to the local microenvironment. Furthermore, virtual screening identified several FDA-approved compounds with high predicted binding affinity for HDGF.

**Conclusion:**

Our study unveils a novel lactylation–HDGF regulatory association that promotes OS progression and modulates the tumor microenvironment. These findings highlight HDGF as a promising prognostic biomarker and therapeutic target, offering new avenues for precision therapy in osteosarcoma.

## 1 Introduction

Osteosarcoma (OS) represents the most common primary malignant tumor of bone and mainly occurs in children and adolescents (1, 2). Although combined treatment strategies involving chemotherapy and surgical resection have improved outcomes for patients with localized disease, individuals with metastatic or recurrent OS continue to experience poor prognosis, with long-term survival rates remaining below 30% (3, 4). A major challenge in OS management is the pronounced intratumoral heterogeneity, which contributes to tumor progression, therapeutic resistance, and metastatic potential (5–8). However, the molecular regulators responsible for shaping this heterogeneity are still not fully understood.

Increasing evidence suggests that post-translational modifications (PTMs) play important roles in cancer development by modulating gene expression and cellular signaling pathways (8–10). Among these modifications, lysine lactylation, a recently identified epigenetic mark derived from lactate metabolism, has attracted growing attention (11). Elevated lactate accumulation within tumors can promote lactylation and subsequently influence transcriptional programs related to cell proliferation, immune regulation, and tumor progression (12, 13). Despite these advances, the distribution and biological significance of the lactylation-related transcriptional landscape in OS remain largely unclear.

Hepatoma-derived growth factor (HDGF) is a multifunctional protein that participates in multiple oncogenic processes, including tumor cell proliferation, angiogenesis, and invasion (14–16). Increased HDGF expression has been linked to poor clinical outcomes in OS (17). Nevertheless, the upstream regulatory mechanisms governing HDGF expression and its potential involvement in lactylation-associated tumor programs have not been investigated (18).

To investigate the potential interplay between lactylation and HDGF in OS, in this study, we applied an integrative framework combining single-cell RNA sequencing, spatial transcriptomics, computational modeling, and experimental validation to explore lactylation-related heterogeneity in OS. Our analysis uncovered a tumor subpopulation with high lactylation activity, genomic instability, and stem-like characteristics. Further network and machine learning analyses identified HDGF as a central candidate regulator within this lactylation-associated transcriptional program. Functional experiments confirmed the oncogenic role of HDGF, and virtual screening suggested several potential HDGF-targeting compounds. Together, these findings reveal a previously unrecognized lactylation–HDGF axis involved in OS progression and provide potential avenues for targeted therapeutic intervention.

## 2 Materials and methods

### 2.1 Acquisition and quality control of single-cell RNA sequencing data

All analyses in this study were based on published public genomic datasets. For bulk RNA-seq-based prognostic modeling and Kaplan–Meier survival analysis, we used the TARGET osteosarcoma cohort, which provides clinically annotated osteosarcoma cases with genomic/transcriptomic profiles and survival information. In addition, GSE21257 was used as an external validation cohort; this dataset contains gene-expression profiles and clinical survival information from 53 OS patients. Kaplan–Meier analyses were performed only in patients with complete overall survival time and survival status, and patients were stratified into high- and low-expression or high- and low-risk groups according to the median expression value or median Risk Score. Single-cell RNA sequencing (scRNA-seq) data were sourced from the Gene Expression Omnibus (GEO) database (accession: GSE162454). This dataset provides transcriptomic profiles of primary, treatment-naïve osteosarcoma tissues from 6 patients, generated using the 10x Genomics Chromium platform, comprising 29,278 cells in total. It comprehensively captures the cellular heterogeneity of the OS ecosystem, including malignant cells as well as stromal and immune cell populations within the tumor microenvironment, making it a valuable resource for characterizing the OS landscape.

### 2.2 Single-cell data processing, clustering, and cell-type annotation

We obtained the pre-filtered gene expression matrix (UMI count matrix) and cell annotations for dataset GSE162454. For further quality control, we used the Seurat (v4.0) package to filter out cells with fewer than 200 or more than 5000 detected genes, and those with a mitochondrial gene percentage exceeding 20%. Data were normalized using the SC Transform method, followed by dimensionality reduction via principal component analysis (Run PCA). Graph-based clustering was performed using FindNeighbors and FindClusters (resolution parameter set to 0.5), and visualization was achieved with RunUMAP. Cell-type identities were assigned using an integrative annotation strategy, rather than relying on single marker genes. This strategy combined three sources of evidence: first, manual annotation based on canonical lineage markers, by reviewing the expression patterns of markers such as ALPL, RUNX2, COL1A1, COL1A2, SP7 (osteoblastic osteosarcoma cells), PECAM1, VWF, KDR with PLVAP (endothelial cells), CD3D, CD3E, TRAC, IL7R (T cells), MS4A1, CD79A, CD79B, CD74 (B cells), MZB1, JCHAIN, IGKC, XBP1 (plasma cells), LYZ, CD68, LST1, C1QA, FCGR3A (myeloid cells), and MS4A2, KIT, TPSAB1 (mast cells); second, cross-validation against previously reported osteosarcoma single-cell signatures; and third, automated annotation using SingleR (v1.6) with the Human Primary Cell Atlas database as a reference, the results of which served as an important guide. Final annotations were determined upon manual review and integrated judgment. Due to the substantial transcriptomic overlap between monocytes and macrophages, reliable distinction based solely on LYZ and CD68 was not feasible; therefore, we adopted a conservative strategy, uniformly annotating cells expressing these shared myeloid markers as a unified “monocyte/macrophage-lineage” population without further subsetting.

### 2.3 Calculation of lactylation score

To quantify the activity of the lactylation-associated transcriptional program at the single-cell level, we calculated a lactylation score for each cell. This score was derived from a curated set of lactylation-associated genes. Specifically, we utilized the lactylation-related gene set compiled by Cheng et al. (19), which comprises 332 genes broadly associated with lactylation states. This established gene set served as the reference for our calculation. Thus, the lactylation score reflects a composite transcriptional output linked to the lactylation metabolic context, rather than being specific to genes with confirmed direct regulation by lysine lactylation marks. The score was computed using the Add Module Score function implemented in the Seurat package. This approach estimates the relative expression of the predefined gene set within each cell while controlling for background gene expression. To visualize the distribution of lactylation activity across the cellular landscape, the lactylation scores were projected onto UMAP embeddings. Differences in lactylation levels among distinct cell populations were further evaluated using ridge plots, allowing for comparison of score distributions across annotated cell types.

### 2.4 High-dimensional weighted gene co-expression network analysis

To delineate tumor-cell-intrinsic transcriptional programs associated with lactylation, we employed the hdWGCNA package (v0.2.0) to construct weighted gene co-expression networks specifically within the malignant osteoblastic cell population. Prior to network construction, non-malignant stromal and immune cells (CAFs, endothelial cells, and various immune lineages) were excluded to ensure the analysis focused on cancer cell-autonomous patterns. For the retained osteoblastic OS cells, the optimal soft-thresholding power (β) was determined using the pick Soft Threshold function to approximate a scale-free network topology. A weighted gene co-expression network was then generated, and gene modules were identified via dynamic tree cutting. Module eigengenes (MEs), representing the first principal component of each module’s expression profile, were calculated and correlated with cellular lactylation scores to identify modules significantly linked to lactylation activity. The module demonstrating the strongest correlation (MAC-M4) was selected for downstream analysis. Finally, network topology analysis was conducted within this lactylation-associated module to identify hub genes possessing central regulatory roles, thereby defining core tumor-intrinsic transcriptional programs driven by lactation.

### 2.5 Machine learning–based prognostic modeling

Candidate genes were identified by intersecting genes from the MAC-M4 module derived from hdWGCNA with the marker genes of the high-lactylation Cluster 4 subpopulation. To select features with prognostic value, univariate Cox proportional hazards regression was initially applied, retaining genes with P < 0.05 as candidate variables for subsequent modeling. For prognostic model construction, bulk RNA-sequencing datasets with matched survival information were sourced from the public TARGET cohort. Patients with complete survival data were included and the dataset was randomly partitioned into a training set (70%) and an internal validation set (30%). An independent external validation was performed using the TARGET cohort. Multiple machine learning algorithms were employed for model building and comparison, including Least Absolute Shrinkage and Selection Operator (LASSO), Elastic Net (Enet), Cox Boost, Random Survival Forest (RSF), and stepwise Cox regression. Model performance was evaluated using the concordance index (C-index) derived from 10-fold cross-validation within the training cohort. The final optimal model was selected based on the highest average C-index across cross-validation folds. For the Elastic Net model, hyperparameters were optimized via grid search. The mixing parameter α was tuned across a range from 0 to 1 (increment = 0.1), and the regularization parameter λ was determined through 10-fold cross-validation aimed at minimizing the partial likelihood deviance. Stepwise Cox regression was conducted using forward selection, with entry and removal criteria set at *P* < 0.05 and *P* > 0.10, respectively. The final prognostic signature was constructed using the “StepCox (forward) + Elastic Net” combination. A risk score for each patient was calculated as a linear combination of the expression levels of the signature genes, weighted by their respective regression coefficients. Patients were then stratified into high- and low-risk groups based on the median risk score. Model performance and predictive accuracy were assessed using Kaplan–Meier survival analysis, time-dependent receiver operating characteristic (ROC) curves, and calibration plots. Decision curve analysis (DCA) was performed to evaluate the clinical net benefit of the prognostic model.

### 2.6 SHAP analysis of prognosis

To improve model interpretability, SHapley Additive exPlanations (SHAP, v0.41) were employed to quantify each feature’s contribution to the risk score (20). SHAP values were computed based on the final Elastic Net–based prognostic model. Global feature importance was assessed by ranking genes according to the mean absolute SHAP values across all samples. SHAP summary plots were generated to visualize both the magnitude and direction of each gene’s impact on model output. Additionally, SHAP dependency plots were used to examine the relationship between gene expression levels and their corresponding SHAP values, thereby uncovering nonlinear effects and potential interaction patterns. Local interpretability was further explored by calculating SHAP values for individual samples, enabling decomposition of the risk score into gene-level contributions. Key driver genes were defined as those consistently exhibiting high SHAP values across multiple analyses. These genes were subsequently subjected to Cox regression and Kaplan–Meier survival analysis to validate their independent prognostic significance.

### 2.7 Prognostic model construction via stepwise Cox and elastic net integration

For the final StepCox (forward) + Elastic Net model, candidate genes were initially identified using forward stepwise Cox regression. The selected genes were subsequently incorporated into an Elastic Net Cox regression framework. The mixing parameter α was tuned across a range of 0 to 1 at intervals of 0.1, while the regularization parameter λ was determined through 10-fold cross-validation, with the optimal value chosen as λ (min) corresponding to the minimum partial likelihood deviance. Model performance across different α–λ combinations was compared based on the cross-validated concordance index (C-index), and the combination yielding the highest performance was retained as the final model. Kaplan–Meier analyses were performed only in patients with complete overall survival time and survival status, and patients were stratified into high- and low-expression or high- and low-risk groups according to the median expression value or median Risk Score.

### 2.8 Spatial transcriptomics analysis

Spatial transcriptomic analysis was performed using a publicly available 10x Genomics Visium osteosarcoma spatial transcriptomics dataset. This dataset provides gene expression profiles with intact spatial context. To analyze the spatial organization of the lactylation-related program, we processed the data using the Seurat package (v4.0). Following normalization and clustering, we annotated the spatial regions into biologically meaningful zones based on a combination of spatial clustering results and matched hematoxylin and eosin (H&E) morphology. This process defined three distinct regions: tumor regions, transition regions (likely representing the invasive margin), and adjacent/normal-like regions.

### 2.9 Virtual screening and molecular docking

To identify potential inhibitors targeting HDGF, virtual screening was performed using the FDA-approved compound subset of the ZINC20 database. Candidate compounds were docked to the three-dimensional structure of HDGF using AutoDock Vina. Binding affinities were calculated based on docking scores, and ligand–protein interactions were further analyzed to evaluate hydrogen bonding, hydrophobic interactions, and structural complementarity within the predicted binding pocket. Compounds were ranked according to their predicted binding energies, and the top candidates were selected for further analysis. Five compounds with the lowest docking energies—Saquinavir, Dihydroergotamine, Montelukast, Ergotamine, and Butanediamide—were chosen for detailed visualization and interaction analysis to assess their potential inhibitory effects on HDGF.

### 2.10 Cell culture

The human OS cell lines 143B and U2OS were obtained from Pricella Biotechnology (Wuhan, China). 143B cells were cultured in Minimum Essential Medium (MEM; Gibco) supplemented with 10% fetal bovine serum (FBS; Gibco) and 1% penicillin–streptomycin (Gibco). U2OS cells were maintained in McCoy’s 5A medium (Gibco) supplemented with 10% FBS and 1% penicillin–streptomycin. Cells were cultured at 37 °C in a humidified incubator with 5% CO_2_. Cell line identity was authenticated by short tandem repeat (STR) profiling, and all experiments were performed using cells within a limited passage number to ensure experimental consistency.

### 2.11 Small interfering RNA knockdown and transfection

Two small interfering RNAs (siRNAs) targeting HDGF were purchased from Gene Pharma (Shanghai, China): si-HDGF-1 (5′-ACCUCUUCCCUUACGAGGAAUCCAA-3′) and si-HDGF-2 (5′-UCCCUUACGAGGAAUCCAAGGAGAA-3′). Cells were transfected using Lipofectamine 3000 (Invitrogen, Carlsbad, CA, USA) according to the manufacturer’s instructions. Briefly, cells were seeded in culture plates and transfected with siRNA–Lipofectamine complexes when they reached appropriate confluency. After transfection, cells were incubated for 48–72 hours, followed by collection for subsequent functional and molecular analyses.

### 2.12 Western blot

Western blot analysis was performed according to previously established protocols. Briefly, total cellular proteins were extracted using lysis buffer and quantified prior to electrophoresis (19 (21). Equal amounts of protein were separated by SDS–PAGE and transferred onto polyvinylidene fluoride (PVDF) membranes. Membranes were then blocked and incubated overnight at 4 °C with primary antibodies. The following primary antibodies were used: anti-HDGF (Cell Signaling Technology, #42105; 1:1000 dilution) and anti-GAPDH (Cell Signaling Technology, #2118; 1:1000 dilution) as a loading control. After washing, membranes were incubated with HRP-conjugated secondary antibodies, including goat anti-mouse IgG (Proteintech, RGAM001; 1:10,000) and goat anti-rabbit IgG (Proteintech, RGAR001; 1:10,000). Protein bands were visualized using an enhanced chemiluminescence (ECL) detection system.

### 2.13 Assessment of cell migration and invasion

The migratory and invasive capacities of OS cells were examined using Transwell chamber assays (Corning, #3422). For invasion experiments, the upper chamber membranes were first coated with Matrigel (BD Biosciences) to simulate the extracellular matrix barrier. Briefly, 143B cells (4 × 10^4^ cells per well) and U2OS cells (5 × 10^4^ cells per well) were suspended in serum-free medium and seeded into the upper compartments of the chambers. The lower chambers contained 500 μL complete medium supplemented with 20% fetal bovine serum (FBS), which served as a chemoattractant. Cells were incubated for 24 hours in migration assays and 48 hours in invasion assays. Following incubation, cells were rinsed with phosphate-buffered saline (PBS), fixed in 4% paraformaldehyde for 20 minutes, and stained using 0.5% crystal violet for 40 minutes. Cells remaining on the upper surface of the membrane were gently removed with cotton swabs. The cells that had migrated or invaded through the membrane were then visualized and quantified under an inverted microscope (Leica Microsystems). All assays were independently performed three times to ensure reproducibility.

### 2.14 *In vivo* xenograft tumor model

All animal experiments were conducted in accordance with institutional guidelines. Six-week-old female BALB/c nude mice (n = 5) were housed under specific pathogen-free (SPF) conditions. To establish the xenograft model, 4 × 10^6^ 143B OS cells suspended in 200 μL serum-free MEM were subcutaneously injected into the right flank of each mouse. Animals were randomly divided into two groups and treated with either negative control siRNA (si-NC) or HDGF-targeting siRNA. The siRNA solution (10 nmol in 0.1 mL saline per tumor) was administered by intratumoral injection every two days for two weeks, resulting in a total of seven injections. Tumor growth was monitored beginning on day 7, and tumor volume was calculated using the formula: V = (length × width²)/2. On day 23, mice were euthanized by gradual CO_2_ inhalation at a fill rate of 30–40% of chamber volume per minute, followed by cervical dislocation to confirm death. Tumors were subsequently excised, photographed, and weighed for downstream analyses.

### 2.15 Immunohistochemical staining

Paraffin-embedded tumor tissues were sectioned at a thickness of 3 μm. Sections were fixed in 4% formaldehyde in PBS, followed by deparaffinization, rehydration, and antigen retrieval according to standard protocols. After blocking, sections were incubated overnight at 4 °C with primary antibodies against Ki67 (ABclonal, A20018) and HDGF (Abcam, ab128921). Following three washes with PBST, the sections were incubated with an HRP-conjugated secondary antibody for 15 minutes at room temperature. Immunoreactivity was visualized using the DAB chromogenic substrate, and nuclei were counterstained with hematoxylin. Finally, the slides were dehydrated, mounted with neutral resin, and examined under a light microscope for histological evaluation.

### 2.16 Inference of copy number variation and genomic instability

To infer large-scale chromosomal copy number variations (CNVs) and assess genomic instability at the single-cell level, we utilized the infer CNV R package (version 3.0.1). Briefly, gene expression counts were normalized and log-transformed. Normal diploid cells (e.g., immune cells from the same sample) were used as reference to define the baseline expression. A moving average approach with a sliding window was then applied across each chromosome to smooth the expression data and infer relative CNV amplitudes. The resulting CNV profiles were used to calculate a genomic instability score for each cell, reflecting the overall deviation from a diploid state.

### 2.17 Statistical analysis

Statistical analyses were performed using R software and GraphPad Prism. Continuous variables that followed a normal distribution were compared using the two-tailed Student’s t-test. Data are presented as mean ± standard deviation (SD) unless otherwise specified. A *p*-value < 0.05 was considered statistically significant.

## 3 Results

### 3.1 Single-cell transcriptomics delineates the cellular architecture and lactylation landscape of OS

**Figure 1:**
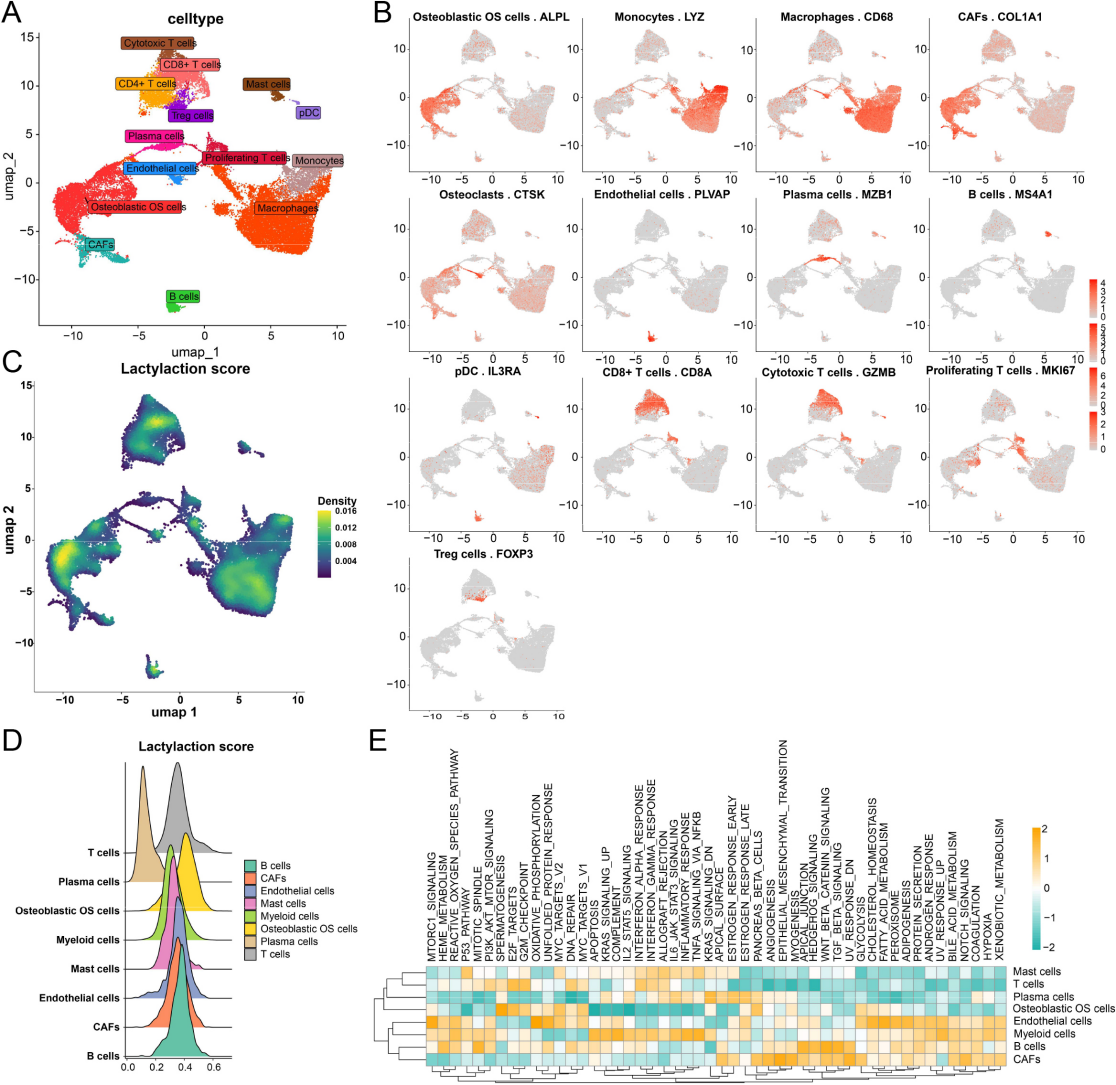
Single-cell transcriptomic immune landscape and lactylation-associated features in osteosarcoma. **(A)** UMAP visualization of single-cell transcriptomes from osteosarcoma tissues showing the major cellular populations, including monocytes, macrophages, plasma cells, B cells, mast cells, and multiple T-cell subsets. **(B)** Feature plots showing the expression of representative marker genes used for cell-type annotation. **(C)** Lactylation scores. **(D)** Ridge plot of immune cells. **(E)** Heatmap of immune cell pathway activity.

To characterize the cellular composition of the OS microenvironment, we performed single-cell transcriptomic profiling and visualized the data using UMAP dimensionality reduction. As shown in **Figure 1A**, OS tissues exhibited marked cellular heterogeneity and were composed of multiple distinct populations, including osteoblastic OS cells, f, endothelial cells, monocytes/macrophages, plasma cells, B cells, mast cells, and several T-cell subsets, including CD8^+^ T cells, cytotoxic T cells, Treg cells, proliferating T cells, and pDC-related immune populations. This clustering pattern highlights the complex multicellular ecosystem of OS and indicates substantial diversity in both stromal and immune compartments.

Cell identity assignment was further supported by the expression of representative marker genes (**Figure 1B**). Osteoblastic OS cells were characterized by high ALPL expression, whereas CAFs showed enriched COL1A1 expression. Myeloid populations were identified by canonical markers such as LYZ in monocytes and CD68 in macrophages. In parallel, PLVAP marked endothelial cells, MZB1 identified plasma cells, MS4A1 defined B cells, IL3RA marked pDCs, and distinct T-cell subsets were annotated by CD8A, GZMB, MKI67, and FOXP3, corresponding to CD8^+^ T cells, cytotoxic T cells, proliferating T cells, and Treg cells, respectively. These marker profiles collectively validate the robustness of the clustering and annotation strategy.

We next assessed the distribution of lactylation-associated transcriptional activity across cellular subsets by calculating a lactylation score for each cell. UMAP projection of the lactylation score demonstrated a non-uniform spatial distribution across the OS cellular landscape (**Figure 1C**), indicating that lactylation-related programs are highly cell-type dependent. Ridge plot analysis further revealed clear differences among major cell populations (**Figure 1D**). In particular, osteoblastic OS cells and CAFs exhibited relatively elevated lactylation scores, whereas lymphoid populations, especially B cells and T cells, generally displayed lower levels. Myeloid and endothelial populations showed intermediate patterns. These findings suggest that lactylation is preferentially activated in tumor and stromal compartments rather than in adaptive immune cells. High-lactylation cell populations were enriched in multiple metabolic and signaling programs, including glycolysis-related pathways, oxidative metabolism, inflammatory signaling, and microenvironment-associated regulatory networks (**Figure 1E**).

### 3.2 Lactylation-associated functional states in OS

**Figure 2:**
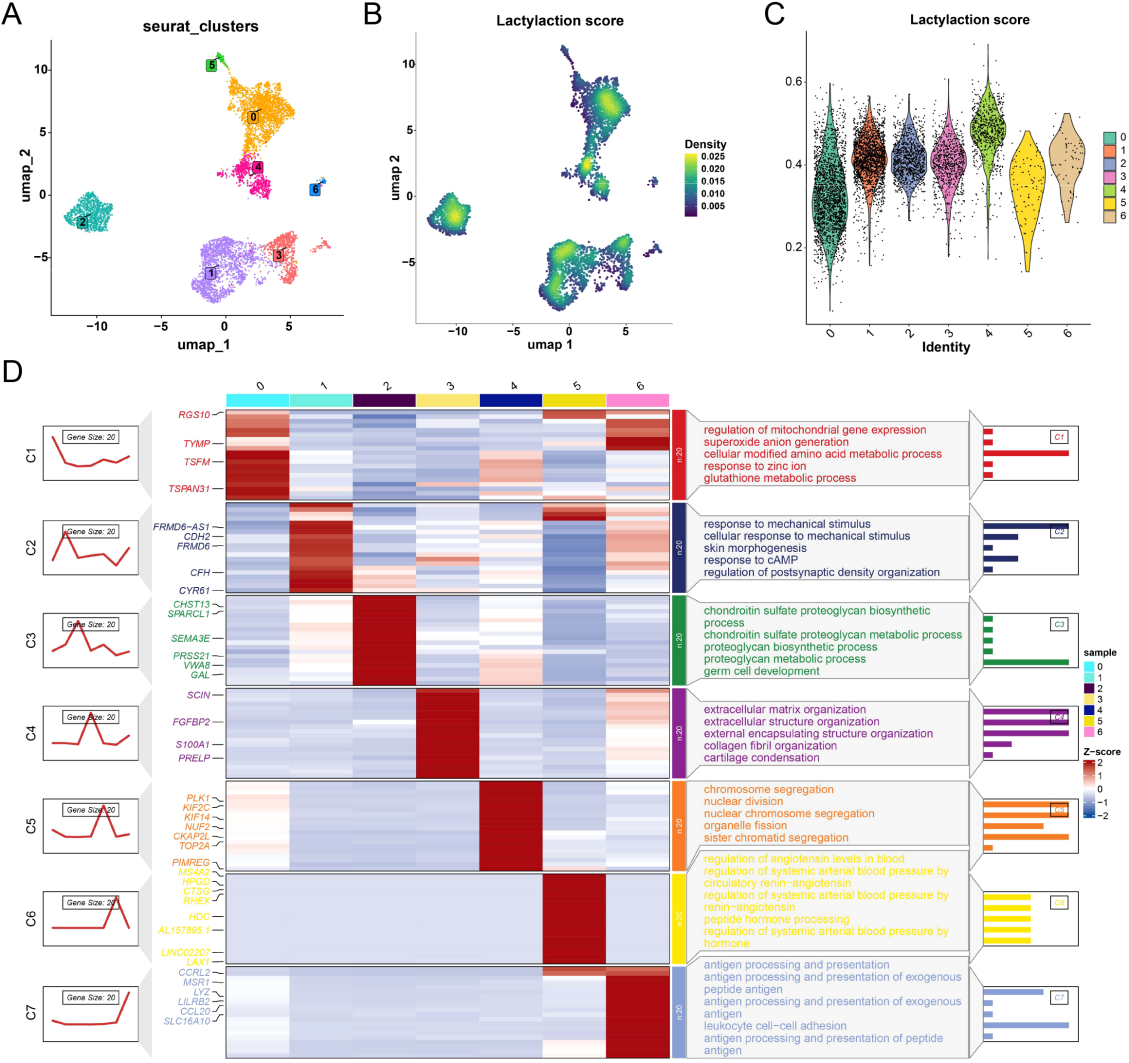
Intratumoral clustering and lactylation-associated functional states of osteosarcoma. **(A)** Osteosarcoma cell clusters identified by unsupervised clustering analysis. Clusters expressing immune-lineage markers (MS4A2, LYZ) were excluded from subsequent tumor-cell-specific analyses. **(B)** Lactylation scores across osteosarcoma cell clusters. **(C)** Violin plots. **(D)** Heatmap showing the relative expression of representative genes across clusters and the corresponding enriched biological processes for each subpopulation.

To further resolve the heterogeneity of OS cells, we performed unsupervised clustering analysis and identified seven transcriptionally distinct subclusters (**Figure 2A**). These clusters showed clear separation on UMAP, indicating substantial intratumoral diversity. We next evaluated the lactylation-associated transcriptional program across OS subpopulations. To capture this, we calculated a “lactylation score” for each cell based on the lactylation-associated gene signature derived from our scRNA-seq analysis. This score reflects a broad transcriptional output associated with the lactylation metabolic state, rather than being restricted to genes with experimentally confirmed direct regulation by lysine lactylation. UMAP projection and violin plot analyses revealed marked inter-cluster heterogeneity in this score (**Figures 2B, C**). Among all clusters, cluster 4 was characterized by the highest lactylation score, whereas the remaining clusters displayed relatively lower or intermediate levels, confirming that elevated lactylation activity is preferentially associated with specific cellular states. Functional enrichment analysis demonstrated pronounced biological divergence among clusters (**Figure 2D**). Notably, cluster 4, the cluster with the highest lactylation score, was strongly enriched for cell cycle–related pathways, including chromosome segregation, nuclear division, and organelle fission, suggesting a close link between elevated lactylation activity and a proliferative cellular state.

### 3.3 The cluster 4 subpopulation exhibits genomic instability, low differentiation, and poor prognostic value

**Figure 3:**
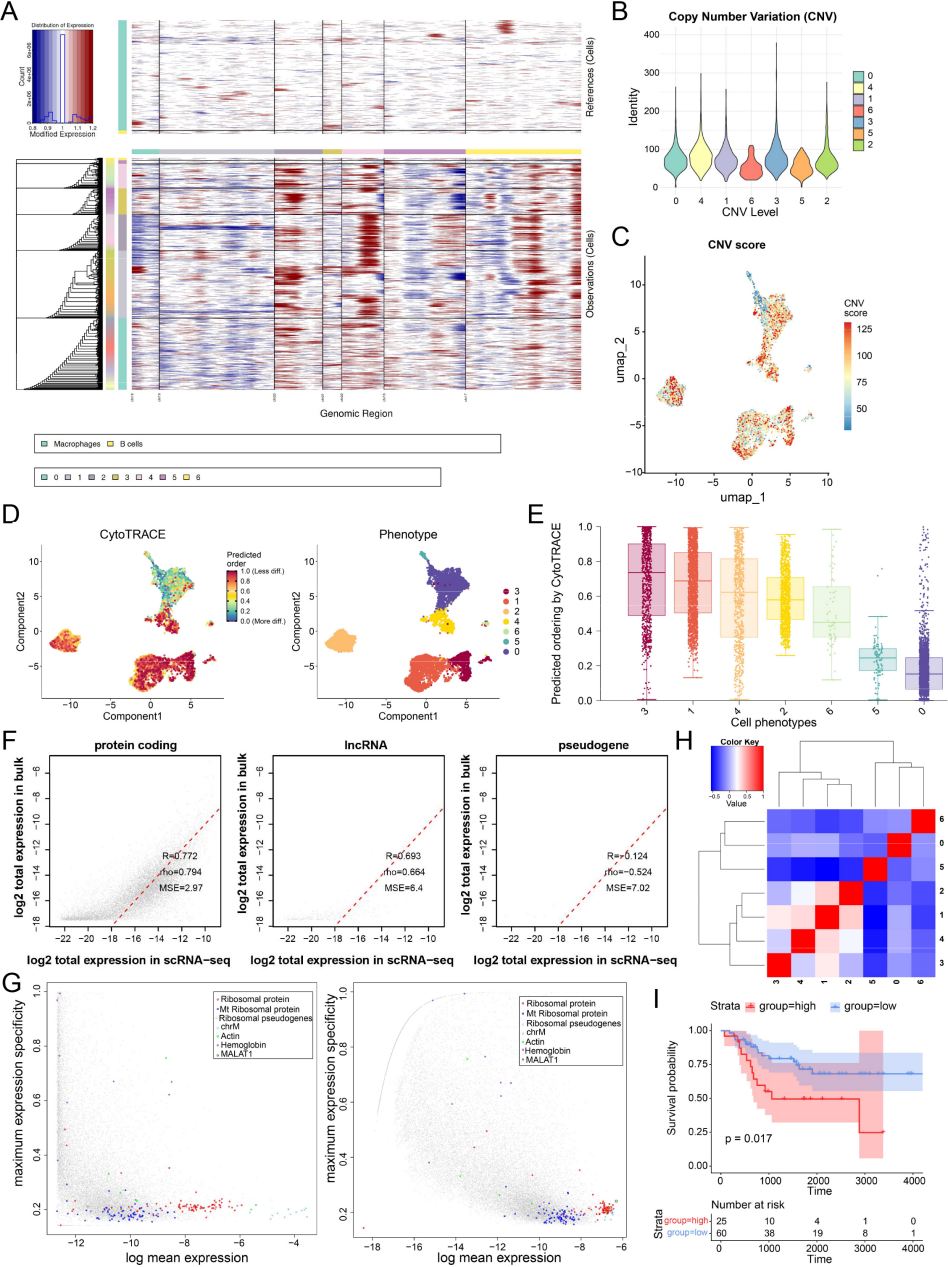
Genomic instability and clinical relevance of osteosarcoma single-cell subpopulations. **(A)** Heatmap of inferred copy number variations (CNVs). **(B)** Violin plot showing the distribution of inferred CNV levels across osteosarcoma cell clusters. **(C)** UMAP visualization of CNV scores. **(D)** CytoTRACE analysis showing the differentiation state of tumor cells and phenotype-based clustering across the osteosarcoma landscape. **(F)** Comparison of CytoTRACE-predicted differentiation order across tumor phenotypic subgroups. **(G)** Correlation between protein-coding genes and pseudogenes. **(H)** Hierarchical clustering. **(I)** Kaplan–Meier survival analysis.

To further define the biological features of OS subpopulations, we first inferred copy number variation (CNV) profiles from single-cell transcriptomic data. Compared with macrophages and B cells, malignant cells exhibited widespread regional CNV alterations, indicating substantial genomic instability (**Figure 3A**). Notably, cluster 4 showed relatively elevated CNV levels and was enriched for high-CNV cells in the UMAP space (**Figures 3B, C**). Tumor cells displayed a continuous developmental spectrum, and phenotype-based mapping identified distinct cellular states across clusters (**Figures 3D, E**). Among them, cluster 4 exhibited higher CytoTRACE scores, indicating a less differentiated and more stem-like phenotype. To validate the robustness of these cellular features, we integrated single-cell and bulk transcriptomic data and observed strong concordance across protein-coding genes, lncRNAs, and pseudogenes (**Figures 3F–H**), supporting the reliability of the deconvolution analysis. Importantly, Kaplan–Meier survival analysis demonstrated that patients with a higher inferred abundance of cluster 4 cells had significantly worse overall survival (**Figure 3I**).

### 3.4 hdWGCNA identifies lactylation-associated co-expression modules in OS cells

**Figure 4:**
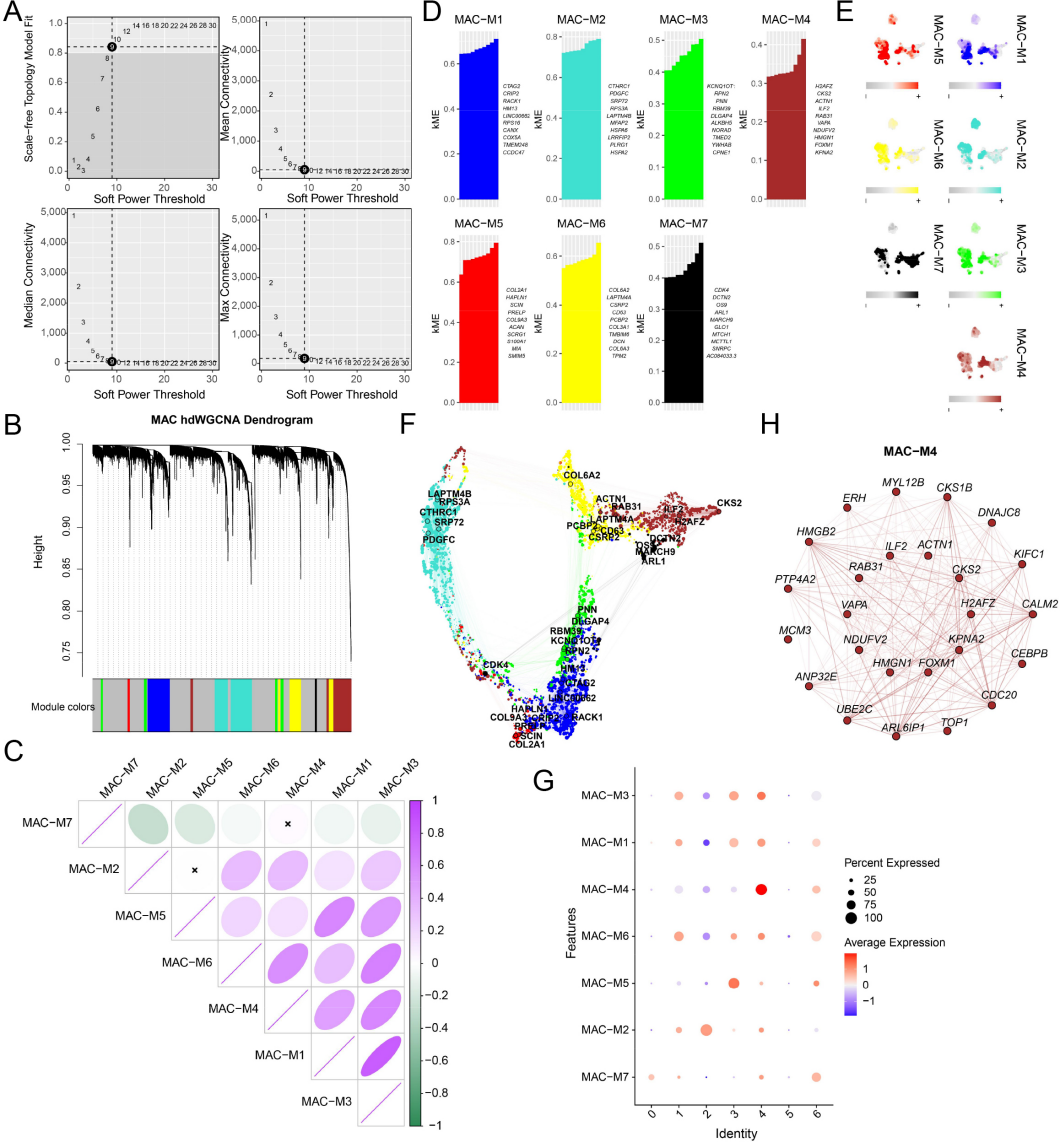
hdWGCNA identifies lactylation-associated co-expression modules in osteosarcoma cells. **(A)** hdWGCNA network construction. **(B)** Co-expression modules. **(C)** Correlation matrix of the identified modules. **(D)** Correlation analysis between each module and lactylation scores. **(E)** UMAPplots showing the spatial distribution of module activity. **(F)** Projection of representative module-associated genes onto the osteosarcoma cell landscape. **(G)** Bubble plot. **(H)** Gene interaction network.

To explore the core regulatory network of the lactylation program within tumor cells, we performed hdWGCNA analysis specifically on the malignant osteoblastic osteosarcoma cell subpopulation. After selecting the optimal soft-thresholding power to approximate scale-free topology (**Figure 4A**), we constructed a co-expression network and identified seven major gene modules (**Figure 4B**). Correlation analysis showed distinct relationships among these modules, indicating substantial functional divergence (**Figure 4C**). Representative genes for each module and their spatial expression patterns are shown in **Figures 4D, E**. Among them, the MAC-M4 module was preferentially enriched in high-lactylation cell subsets and displayed stronger activity than other modules (**Figures 4F, G**), suggesting a close association with lactylation-related transcriptional states. Network analysis of MAC-M4 further identified a densely connected hub centered on genes such as TOP1, CKS2, and HJURP (**Figure 4H**).

### 3.5 Machine learning–based prognostic modeling identifies a risk signature associated with a lactylation-related transcriptional program

**Figure 5:**
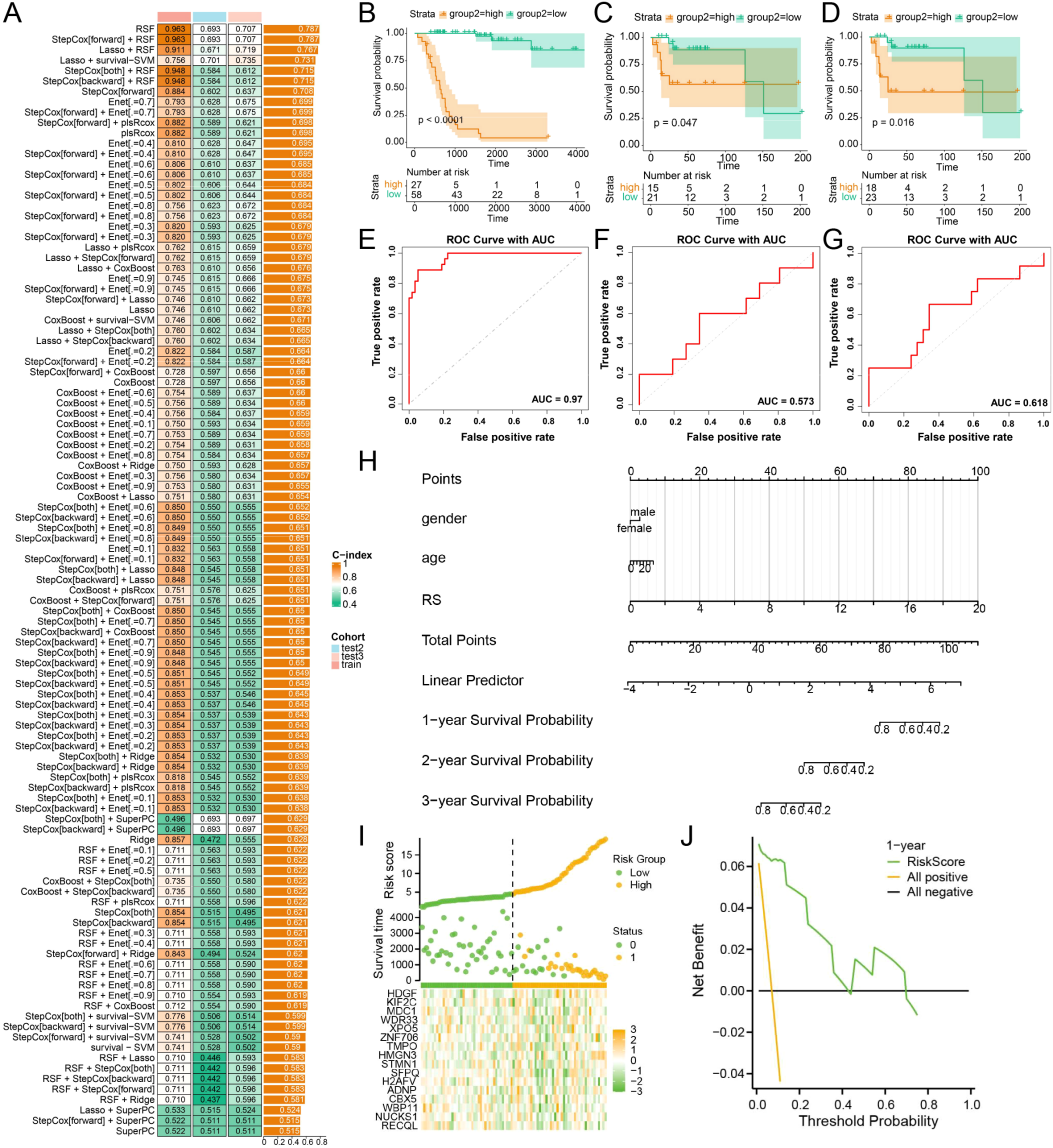
Lactylation-associated prognostic signature in osteosarcoma. **(A)** Heatmap comparing the performance of multiple machine-learning models across the training and validation cohorts. **(B–D)** Kaplan–Meier survival curves. **(E–G)** Time-dependent ROC curves evaluating the predictive accuracy of the risk model in the training and validation cohorts. **(H)** Nomogram integrating the risk score. **(I)** Distribution of risk score. **(J)** Decision curve analysis.

Comparative analysis showed that the StepCox (forward) + Enet model achieved the best overall performance, with the highest C-index across the training and validation cohorts (**Figure 5A**). Patients stratified into the high-risk group had significantly worse survival than those in the low-risk group in the training and external validation cohorts (**Figures 5B–D**), indicating stable prognostic discrimination. ROC analysis further supported the predictive value of the model, with strong performance in the training set and moderate but retained accuracy in the validation cohorts (**Figures 5E–G**). To facilitate clinical application, we established a nomogram incorporating the risk score and clinical variables to predict 1-, 2-, and 3-year survival probabilities (**Figure 5H**). Risk distribution analysis showed that increasing risk scores were associated with shorter survival time and higher mortality, accompanied by distinct expression patterns of model genes (**Figure 5I**). In addition, decision curve analysis demonstrated that the model provided a measurable net clinical benefit across a range of threshold probabilities (**Figure 5J**).

### 3.6 SHAP analysis identifies key drivers of the lactylation-associated prognostic signature

**Figure 6:**
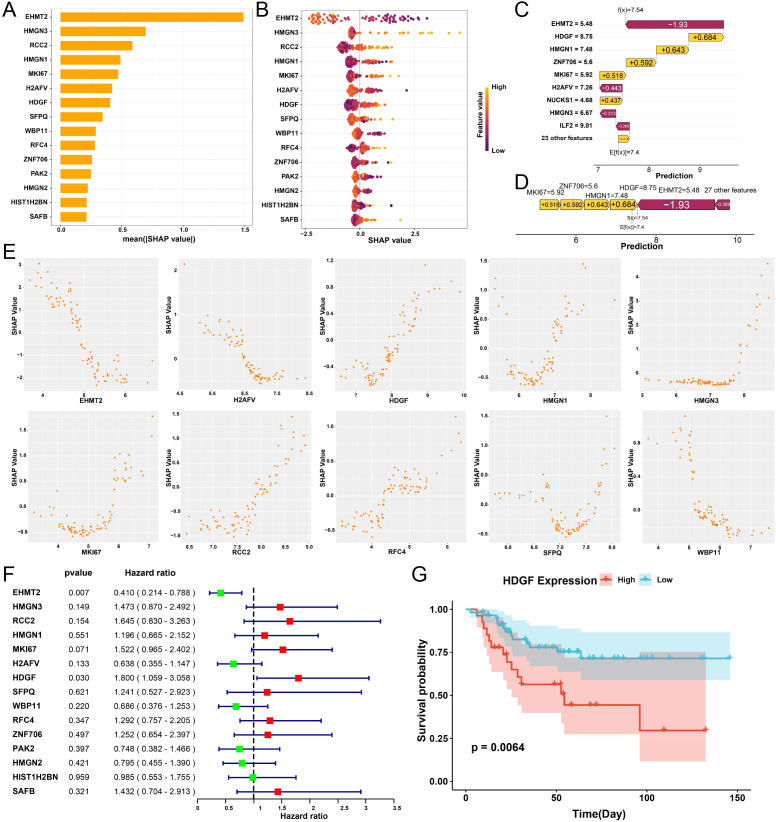
SHAP-based interpretation of machine-learning model features and identification of core prognostic genes. **(A)** Bar plot showing the mean absolute SHAP values of model features, ranking their overall contribution to risk prediction. **(B)** SHAP summary plot illustrating the distribution, direction, and magnitude of each feature’s effect on model output. **(C)** Waterfall plot showing the contribution of individual genes to the predicted risk score for a representative sample. **(D)** Force plot. **(E)** SHAP dependence plots showing the relationship between gene expression and SHAP values for representative model genes, highlighting their contribution patterns to risk prediction. **(F)** Forest plot of Cox regression analysis showing the hazard ratios and 95% confidence intervals of model genes associated with survival. **(G)** Kaplan–Meier survival curve comparing overall survival between patients with high and low HDGF expression.

To improve the interpretability of the prognostic model, we performed SHapley Additive exPlanations (SHAP) analysis to quantify the contribution of individual genes to risk prediction. Ranking by mean absolute SHAP values identified EHMT2, HMGN3, RCC2, HMGN1, MKI67, H2AFV, and HDGF as the major contributors to model output (**Figure 6A**). SHAP summary plots further illustrated the direction and magnitude of each feature effect, showing that high expression of HDGF, HMGN1, HMGN3, and RCC2 was associated with increased predicted risk, whereas elevated EHMT2 expression was linked to reduced risk (**Figure 6B**). Both local explanation and feature attribution analyses highlighted HDGF as a key determinant of model prediction (**Figures 6C, D**). SHAP dependence plots showed coordinated relationships between HDGF and several risk-associated genes, including HMGN1, HMGN3, RCC2, and MKI67, suggesting that these genes may act cooperatively within the lactylation-related malignant program (**Figure 6E**). Cox regression analysis further supported the prognostic relevance of selected model genes. In particular, HDGF was significantly associated with poorer survival, whereas EHMT2 showed a protective association (**Figure 6F**). Consistent with this result, Kaplan–Meier analysis demonstrated that patients with high HDGF expression had significantly worse overall survival than those with low expression (**Figure 6G**). Given its central hub status in the hdWGCNA co-expression network and its established oncogenic roles (14, 22), HDGF was selected as the primary candidate for subsequent functional validation and mechanistic investigation.

### 3.7 Spatial transcriptomics reveals regional enrichment of lactylation and HDGF in OS tissues

**Figure 7:**
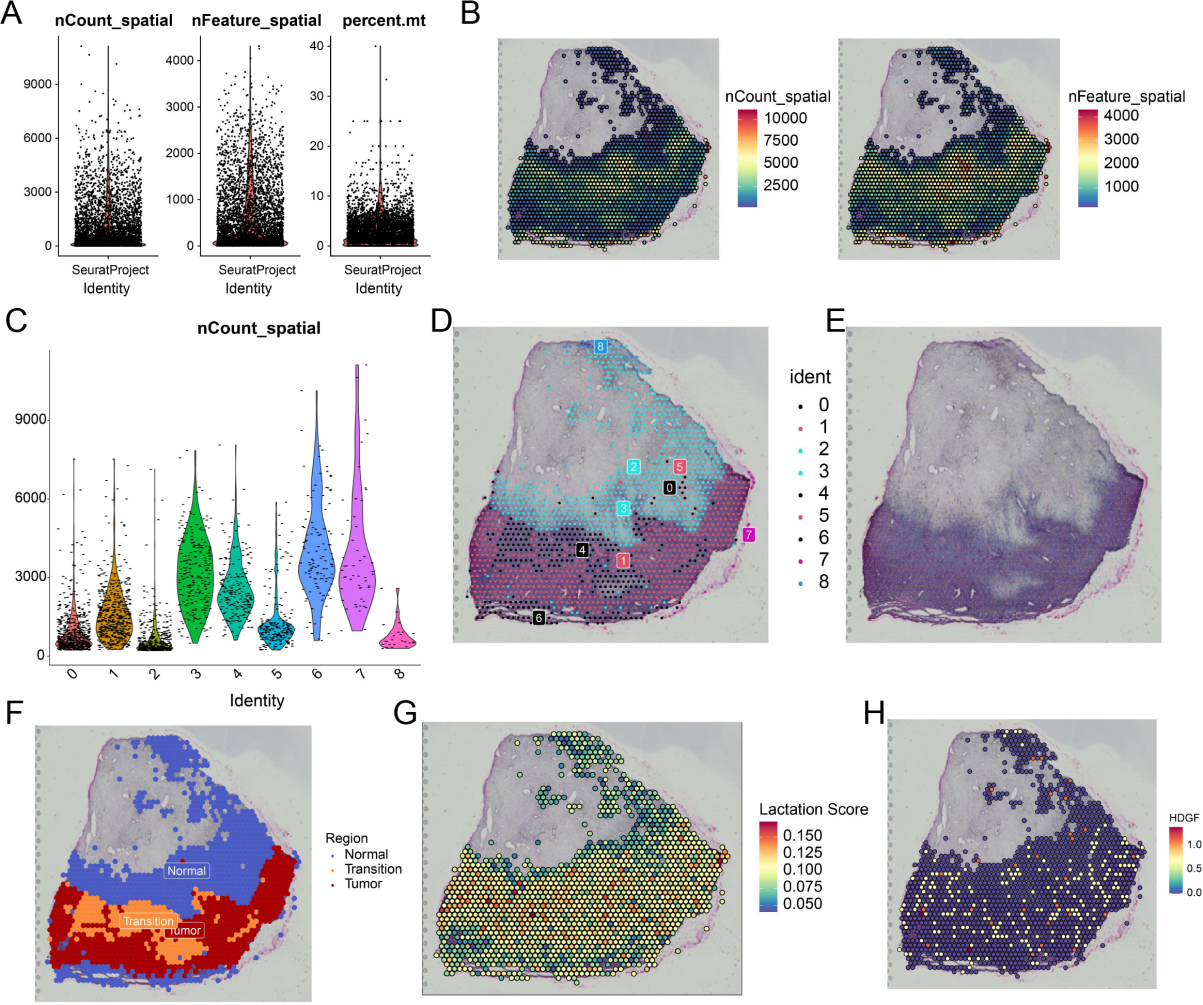
Spatial transcriptomics analysis of osteosarcoma reveals spatial co-localization of lactylation features and HDGF expression. **(A)** Quality-control metrics. **(B)** Spatial distribution of nCount_spatial and nFeature_spatial across the tissue section. **(C)** Violin plot showing the distribution of nCount_spatial across spatial clusters. **(D)** Spatial clustering analysis identifying distinct transcriptional domains within the osteosarcoma tissue section. **(E)** H&E-stained tissue morphology. **(F)** Region annotation. **(G)** Spatial projection of lactylation scores across the tissue section. Color intensity represents the z-score normalized activity. **(H)** Spatial expression pattern of HDGF in the osteosarcoma tissue section. Color intensity represents the log-normalized expression counts.

To validate the single-cell findings *in situ*, we performed spatial transcriptomic profiling of OS tissue sections. Quality control metrics, including nCount_spatial, nFeature_spatial, and percent.mt, indicated good data quality (**Figure 7A**). Spatial distribution of transcript counts and detected features revealed substantial regional heterogeneity across the tissue section (**Figures 7B, C**). Unsupervised clustering identified nine spatial domains with distinct transcriptional patterns (**Figures 7D, E**). Based on histological features, the tissue was further annotated into normal, transition, and tumor regions (**Figure 7F**). Notably, the tumor region displayed a distinct molecular profile compared with the non-tumor areas. Spatial mapping showed that lactylation scores were predominantly enriched in tumor regions (**Figure 7G**). Consistent with this pattern, HDGF expression was also concentrated in the same spatial areas (**Figure 7H**). The concordant spatial distribution of elevated lactylation and HDGF expression supports a close association between HDGF and lactylation-enriched malignant regions in OS.

### 3.8 Comprehensive pan-cancer profiling of HDGF: expression, prognostic significance, genetic alterations, epigenetic regulation, immune correlations, and drug sensitivity

To complement and contextualize our core findings on HDGF in osteosarcoma, we conducted an exploratory analysis of HDGF expression and function across cancers. First, we assessed HDGF transcript levels using The Cancer Genome Atlas (TCGA) dataset, which revealed significant upregulation of HDGF in 16 cancer types compared to adjacent normal tissues (**Supplementary Figure 1A**). Integration with GTEx data further identified HDGF overexpression in 26 cancer types (**Supplementary Figure 1B**). Analysis of paired samples from TCGA confirmed HDGF was highly expressed in 14 cancer types (**Supplementary Figure 1C**). Prognostic evaluation indicated that elevated HDGF expression was associated with poorer outcomes in various tumors, including hepatocellular carcinoma (**Supplementary Figures 1D–F**). Next, we interrogated the genomic alteration landscape of HDGF. Mutation data from cBioPortal showed that HDGF mutation frequencies were highest in cholangiocarcinoma, hepatobiliary cancer, and breast cancer (**Supplementary Figure 2A**). Furthermore, we observed that copy number variations (CNVs) of HDGF could modulate its expression, correlating with increased or decreased transcript levels in different cancers (**Supplementary Figures 2B, C**). Given the key role of DNA methylation in cancer epigenetics, we investigated its regulation on HDGF. Significant differences in HDGF DNA methylation levels between tumor and normal tissues were observed in 5 cancer types (**Supplementary Figures 3A, B**). We performed analysis using the TIMER database to explore the relationship between HDGF and the tumor immune microenvironment. This revealed significant associations between HDGF expression and the abundance of various immune cell types across 30 cancer types. Spearman correlation analysis using multiple algorithms demonstrated significant, albeit variable, correlations between HDGF expression and levels of different tumor-infiltrating immune cells in pan-cancer (**Supplementary Figures 4A, B**), underscoring a tight link between HDGF and the immune landscape. Finally, analysis of drug sensitivity indicated that HDGF expression was negatively correlated with sensitivity to multiple chemotherapeutic agents, including austocystin D, BMS−536924, canertinib, and insitinib (**Supplementary Figure 5**).

### 3.9 HDGF knockdown suppresses malignant phenotypes of OS cells *in vitro* and *in vivo*

**Figure 8:**
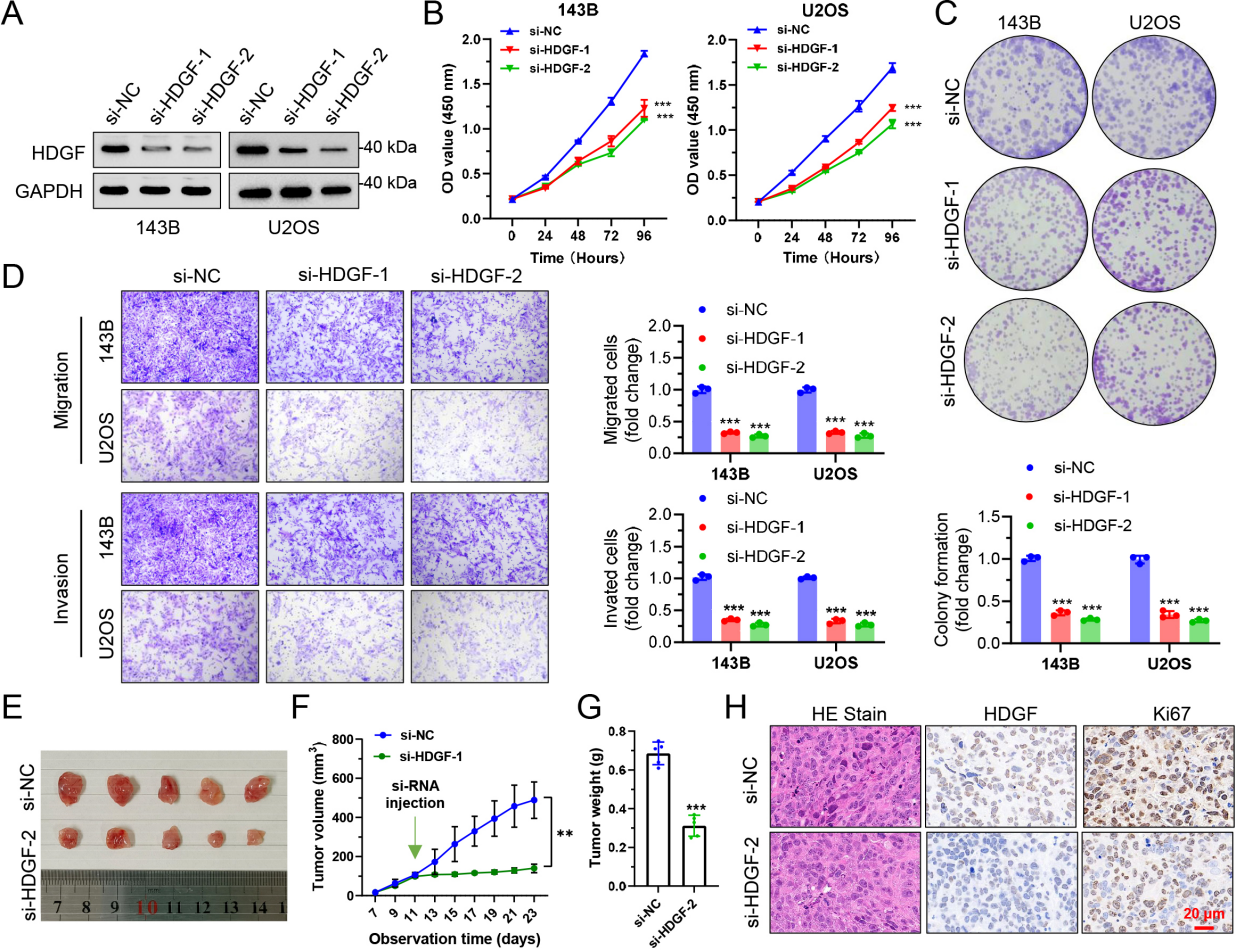
Functional validation of HDGF in OS *in vitro* and *in vivo*. **(A)** Western blot. **(B)** CCK-8 assays. **(C)** Colony formation assay. **(D)** Transwell migration and invasion assays. **(E)** xenograft tumors derived from control and HDGF-knockdown cells. **(F)** Tumor growth curves. **(G)** Tumor weights in the control and HDGF-knockdown groups. **(H)** H&E, HDGF, and Ki67 staining of xenograft tumor sections. **P < 0.01, ***P < 0.001.

To validate the functional role of HDGF in OS, we performed loss-of-function experiments in 143B and U2OS cells. Western blot confirmed effective knockdown of HDGF after siRNA transfection in both cell lines (**Figure 8A**). HDGF silencing significantly inhibited cell proliferation, as shown by reduced growth curves in CCK-8 assays (*P* < 0.001) (**Figure 8B**). Consistently, colony formation capacity was markedly decreased following HDGF depletion (**Figure 8C**). Transwell assays further demonstrated that knockdown of HDGF suppressed both migratory and invasive abilities in 143B and U2OS cells (**Figure 8D**). We next assessed the *in vivo* effect of HDGF depletion using a xenograft model. Tumors derived from HDGF-silenced cells grew more slowly than controls, resulting in smaller tumor size, reduced tumor volume, and lower final tumor weight (**Figures 8E–G**). Histological and immunohistochemical analyses further showed reduced Ki67 staining in the HDGF-knockdown group, indicating impaired proliferative activity *in vivo* (**Figure 8H**).

### 3.10 Virtual screening and molecular docking identify candidate small-molecule inhibitors of HDGF

**Figure 9:**
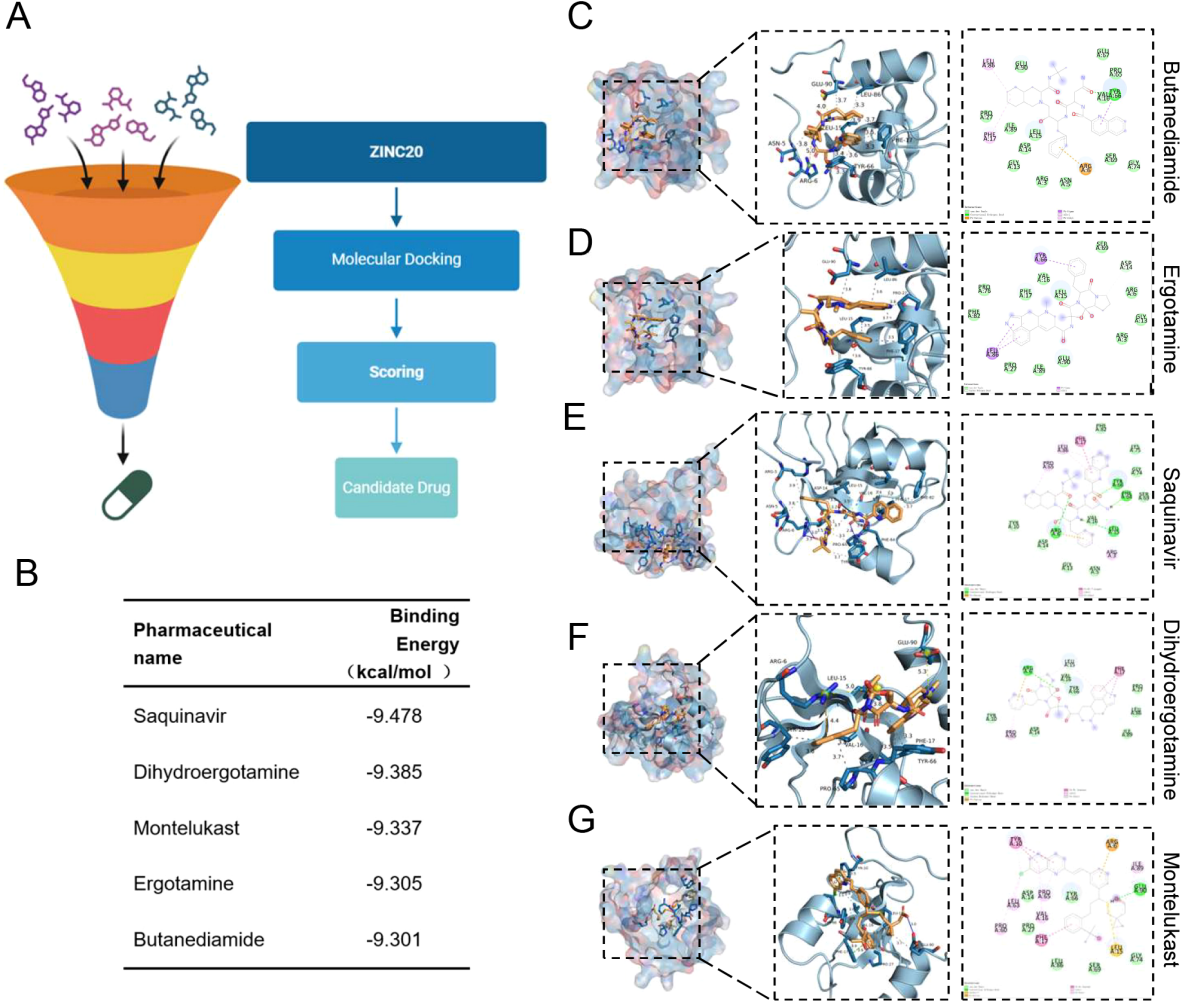
Virtual screening and molecular docking of drugs targeting HDGF. **(A)** Workflow of virtual screening and molecular docking. **(B)** Top-ranked candidate compounds. **(C–G)** Molecular docking models showing the binding modes of Butanediamide **(C)**, Ergotamine **(D)**, Saquinavir **(E)**, Dihydroergotamine **(F)**, and Montelukast **(G)** with HDGF.

To explore potential therapeutic agents targeting HDGF, we performed virtual screening of the ZINC20 database followed by molecular docking analysis (**Figure 9A**). Based on docking scores, five compounds showed the strongest predicted binding affinity for HDGF, including Saquinavir, Dihydroergotamine, Montelukast, Ergotamine, and Butanediamide (**Figure 9B**). All five candidates exhibited favorable binding energies, suggesting stable interactions with the HDGF protein. Docking analysis further showed that these compounds occupied a similar binding pocket on HDGF and formed multiple non-covalent interactions with surrounding residues (**Figures 9C–G**). Among them, Saquinavir displayed the lowest binding energy, indicating the strongest predicted affinity. Dihydroergotamine and Montelukast also showed stable binding patterns, whereas Ergotamine and Butanediamide demonstrated slightly weaker but still favorable interactions.

## 4 Discussion

OS is characterized by profound cellular heterogeneity and a complex tumor microenvironment that integrates metabolic, genetic, and immune regulatory processes. However, the mechanisms linking metabolic reprogramming to tumor heterogeneity and immune modulation remain incompletely understood (23). To address this, we employed an integrative multi-omics strategy combining single-cell and spatial transcriptomics with machine learning–based modeling to systematically map lactylation-associated programs in OS. Our analysis leveraged a publicly curated gene set of 332 lactylation-related genes to infer lactylation activity. This approach delineated a distinct, highly malignant tumor subpopulation (Cluster 4) defined by this inferred high lactylation-related transcriptional score, heightened genomic instability, and stem-like features. Critically, the abundance of this subpopulation correlated strongly with poor patient prognosis, underscoring the clinical relevance of lactylation-driven oncogenic programs.

A key discovery is the association between a specific lactylation-enriched transcriptional state and core hallmarks of malignancy. Cells within this state displayed increased CNV burden and a less differentiated phenotype, suggesting lactylation may fuel tumor cell plasticity and progression (8, 24). It is noteworthy that the lactylation-related gene set includes proliferation-associated markers such as MKI67, implying that the high-scoring subpopulation likely represents a broader, aggressive tumor state driven by lactylation, characterized by high proliferative potential, rather than a singular modification process. Increasing evidence indicates that lactylation serves as a key molecular bridge between metabolic remodeling and epigenetic regulation in cancer (25). Elevated glycolytic activity leads to excessive lactate accumulation in the tumor microenvironment, which can induce lysine lactylation and subsequently reshape transcriptional programs governing cell proliferation, metabolic adaptation, and immune regulation (19, 20). Our spatial transcriptomic data provide direct *in situ* evidence for this concept, showing that regions of high lactylation scores spatially coincide with tumor territories, implying lactylation helps establish and maintain localized malignant niches (26, 27).

Beyond cell-intrinsic effects, lactylation likely influences the immune microenvironment. Tumor-derived lactate is known to suppress cytotoxic T-cell function and promote pro-tumorigenic macrophage polarization (28, 29). Consistent with an immunomodulatory role, our single-cell analysis revealed that lactylation activity is preferentially enriched in tumor and stromal compartments, while adaptive immune cells (B and T cells) exhibit lower levels. This compartment-specific distribution suggests that lactylation may contribute to an immunosuppressive milieu by altering the metabolic landscape of the tumor niche. Prior studies showing lactylation can directly regulate immunosuppressive gene expression in macrophages further support this potential link in OS (30, 31).

Within this lactylation-associated network, HDGF emerged a central candidate. While the direct regulatory mechanism linking lactylation to HDGF remains to be established, our data strongly support HDGF as a key downstream executor of this malignant program. Although HDGF has established roles in promoting proliferation, angiogenesis, and metastasis in various cancers (32, 33), its connection to lactylation-mediated regulation was previously unknown. Our hdWGCNA identified HDGF as a hub gene within a lactylation-correlated module enriched for cell cycle and proliferation pathways (34). This finding was reinforced by SHAP analysis of our prognostic model, which highlighted HDGF as a dominant feature driving high-risk prediction. Notably, the suppression of cell proliferation upon HDGF knockdown aligns with the enrichment of cell cycle–related pathways specifically in the high-lactylation subpopulation (Cluster 4). Functional validation confirmed its pivotal role, as HDGF knockdown potently suppressed OS cell proliferation, migration, invasion, and *in vivo* tumor growth. These results position HDGF as a critical downstream effector executing lactylation-associated proliferative and invasive programs.

Our pan-cancer analysis further contextualizes the role of HDGF. We observed that HDGF is frequently overexpressed and associated with unfavorable prognosis across a wide spectrum of cancers, underscoring its conserved function as a potent oncogenic driver. This broad relevance highlights the potential translational value of targeting HDGF. Importantly, while HDGF appears to be a common node in cancer progression, the lactylation-associated regulatory axis identified in our OS model suggests a context-specific upstream mechanism. In OS, metabolic reprogramming and the resultant lactylation may serve as a key switch that activates or amplifies this otherwise widely utilized oncogenic pathway, thereby contributing to the distinct aggressiveness and heterogeneity of this malignancy. Notably, HDGF may also interface with immune modulation (35). Previous reports indicate HDGF can influence inflammatory signaling, extracellular matrix remodeling, and angiogenesis—processes integral to shaping the immune microenvironment (36). Thus, the lactylation-associated HDGF regulation axis we identified may represent a key regulatory pathway that coordinately drives tumor proliferation and immune microenvironment remodeling in OS (37).

From a translational perspective, we performed an initial exploratory computational drug targeting study. Our virtual screening and molecular docking against the ZINC20 database identified several compounds, including FDA-approved drugs like Saquinavir, Dihydroergotamine, and Montelukast, as high-affinity predicted binders of HDGF. It is important to emphasize that these are computational predictions; their actual efficacy in inhibiting HDGF function and suppressing OS growth remains to be experimentally validated. Nonetheless, drug repurposing offers a strategic avenue for accelerating therapy development in OS, where targeted options are limited (37, 38). Among the candidates, Saquinavir showed the strongest predicted binding affinity, thereby highlighting it as a prioritized candidate for future functional and preclinical testing. This work thus frames a clear and promising future direction for validating HDGF-targeted therapeutic strategies in OS.

Several study limitations should be noted. First, while we establish a strong association, the precise molecular mechanism by which lactylation is linked to HDGF—whether through direct modification of the HDGF protein, lactylation-dependent transcriptional activation, or other indirect means—requires further elucidation. Second, the prognostic model, while robust across tested datasets, necessitates prospective validation in larger, independent clinical cohorts. Third, the candidate inhibitors identified computationally require experimental confirmation of their binding specificity, efficacy, and safety in OS models. Fourth, the inference of CNV and genomic instability scores in this study was derived from scRNA-seq data using bioinformatic tools. While this approach provides a scalable means to assess intratumoral heterogeneity, it is an indirect method based on transcriptional signals, and its resolution may be lower than that of direct genomic approaches such as whole-genome sequencing. Future studies integrating matched genomic data would be valuable to confirm these findings.

## 5 Conclusion

This study delineates the lactylation-related transcriptional landscape within the osteosarcoma (OS) ecosystem through an integrative multi-omics approach. We identified a clinically adverse tumor subpopulation marked by high lactylation activity, genomic instability, and stem-like features. Multi-angle evidence — including spatial co-localization, prognostic model interpretation (SHAP analysis), and functional validation — collectively nominate HDGF as a pivotal candidate effector associated with the lactylation-related program, though its direct causal role remains to be fully elucidated, linking metabolic reprogramming to aggressive tumor phenotypes and immune microenvironment remodeling. Our work not only establishes a direct association between a metabolite-derived modification and core malignant features (proliferation, genomic instability, poor differentiation) in OS but also provides spatial evidence for the co-enrichment of a specific epigenetic state and its downstream oncogene. Furthermore, the identification of FDA-approved compounds with high predicted binding affinity for HDGF via virtual screening provides preliminary computational leads and candidate molecules for future exploration of HDGF-directed therapeutic strategies, which will require substantial subsequent experimental validation. These findings advance our understanding of metabolic-epigenetic-immune crosstalk in OS and offer a concrete rationale for developing targeted interventions.

## Data Availability

The original contributions presented in the study are included in the article/supplementary material. Further inquiries can be directed to the corresponding author/s.
