# Supplementary material for: Lactylation-driven metabolic reprogramming promotes osteosarcoma malignancy via HDGF-mediated proliferation and immune modulation
Source: Front Immunol. 2026 May 20;17:1836254. doi: 10.3389/fimmu.2026.1836254 (PMC13229866; doi:10.3389/fimmu.2026.1836254)
Supplement: Supplementary file 1 [file DataSheet1.docx]

**Lactylation-driven metabolic reprogramming promotes osteosarcoma malignancy via HDGF-mediated proliferation and immune modulation**


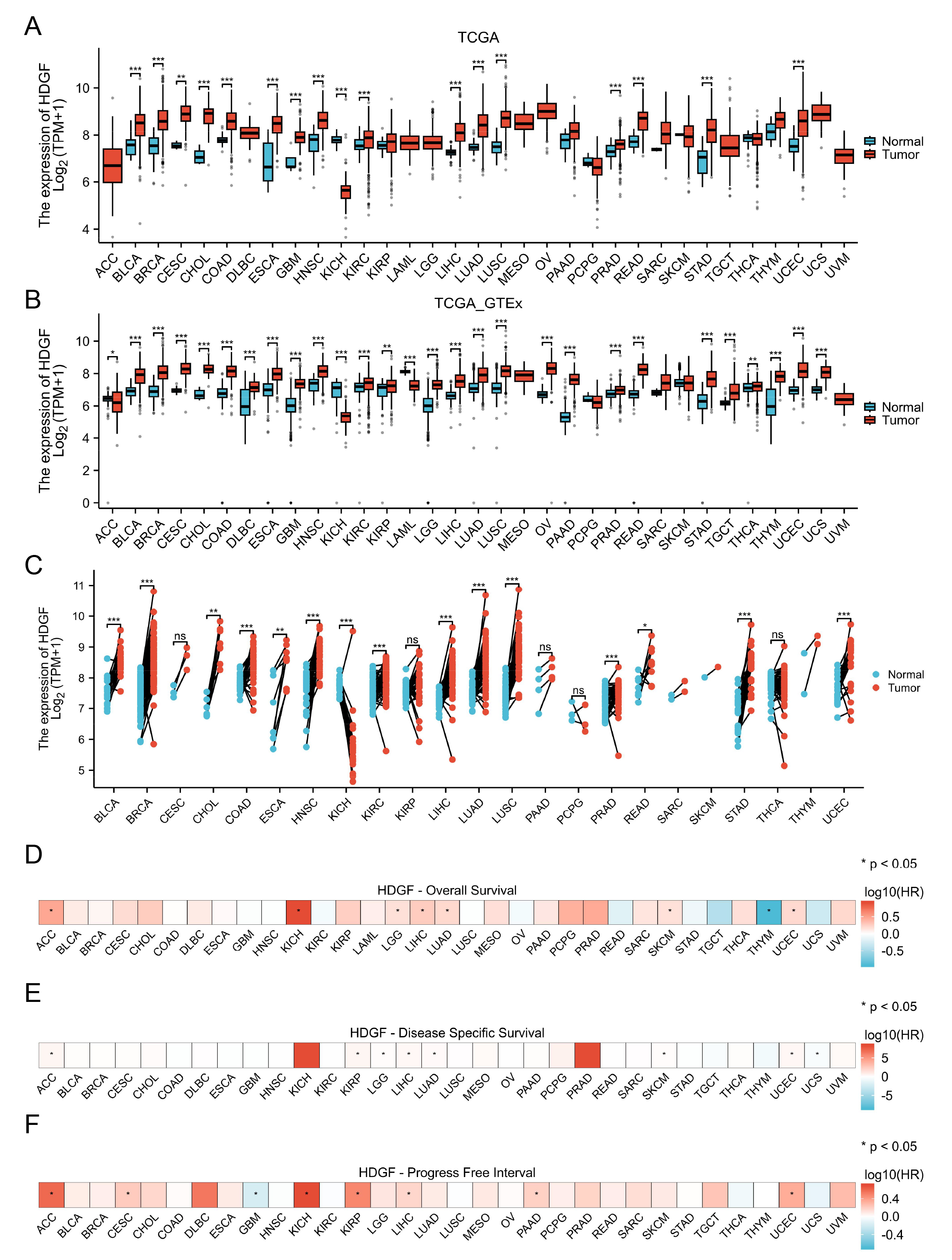


**Figure S1 Expression analysis of SFR1 across human cancers.** (A) Transcript levels of HDGF in tumor tissues from the TCGA database. (B) Transcript levels of HDGF from the TCGA and GTEx databases (Tumor vs. Normal). (C) A thorough investigation of HDGF expression in tumor tissues alongside their respective adjacent normal tissues was carried out, employing data sourced from the TCGA. (D-F) Heat map of prognostic analysis of HDGF in pan-cancer. ns *P*>0.05, * *P* <0.05, ** *P* <0.01, *** *P* <0.001.

**
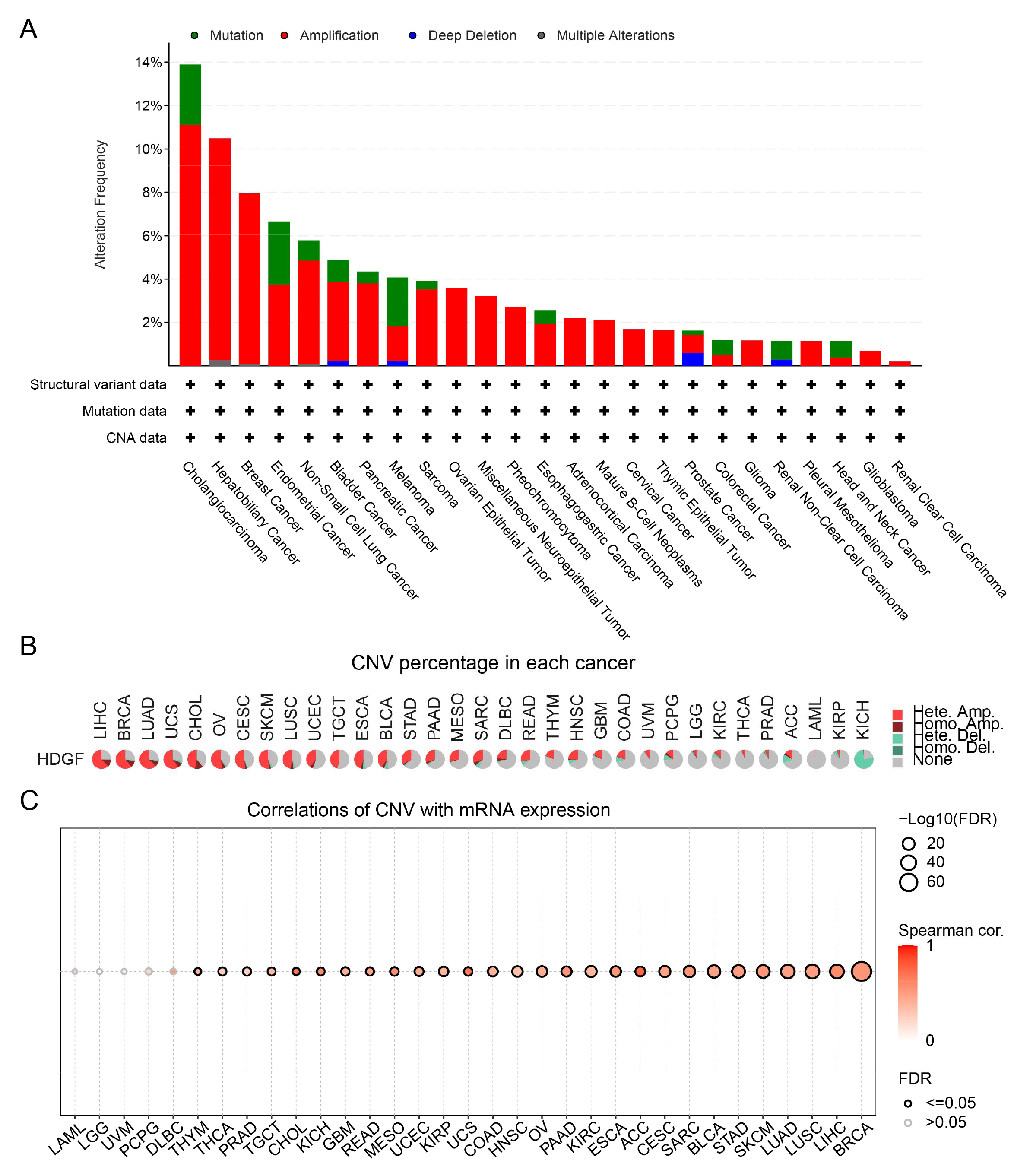
**

**Figure S2 Genomic landscape of HDGF in human cancers.** (A) The mutation frequency of HDGF across different cancer types from the cBioPortal database. (B) The impact of HDGF copy number variations (CNVs) on its mRNA expression levels in tumors. (C) Correlation analysis between HDGF expression and CNVs.

**
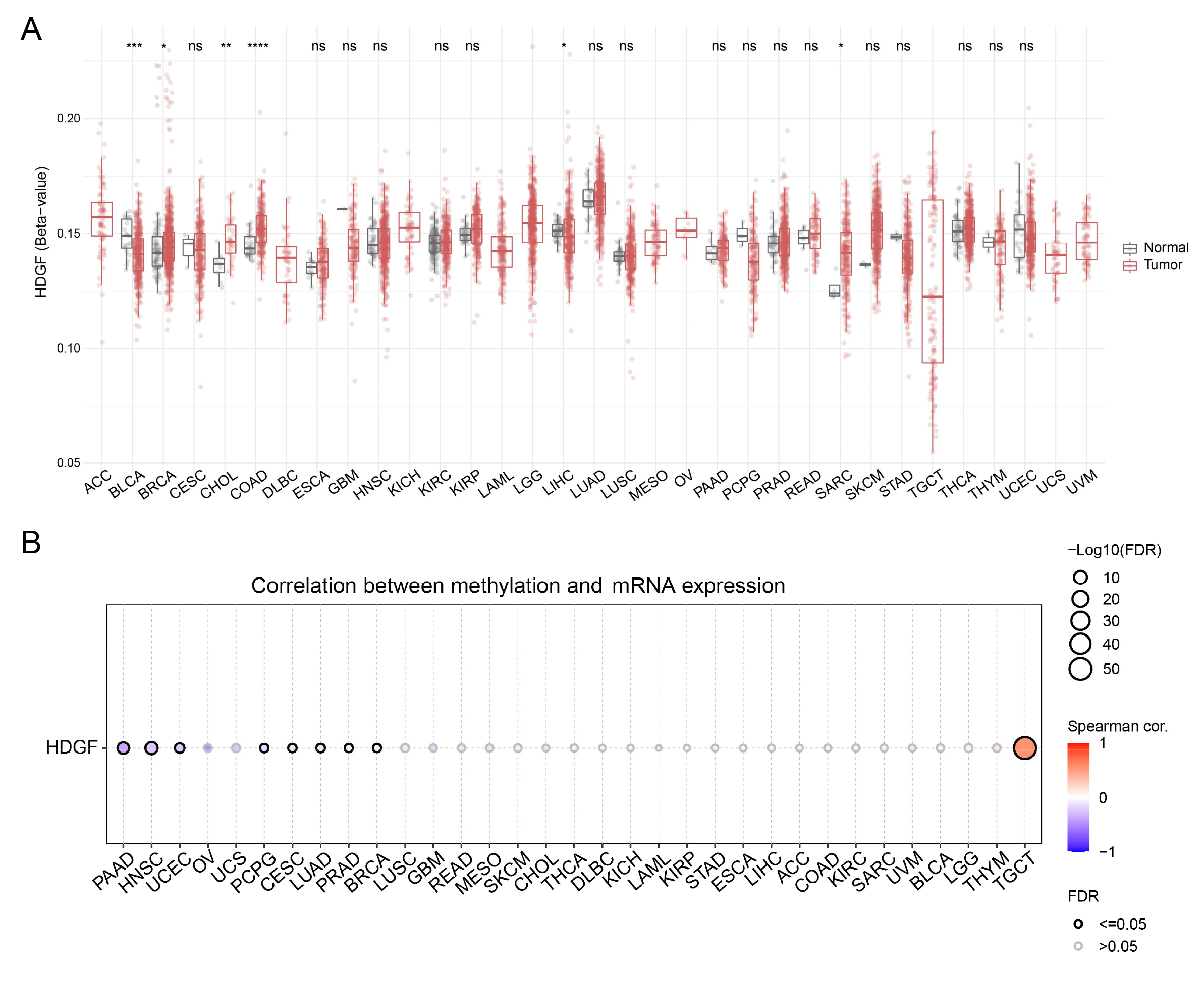
**

**Figure S3 Epigenomic landscape of HDGF in human cancers.** (A) Differential DNA methylation levels of the HDGF gene promoter between tumor and normal tissues in seven cancer types. (B) Correlation analysis between HDGF expression and DNA methylation levels. ns *P*>0.05, * *P* <0.05, ** *P* <0.01, *** *P* <0.001.

**
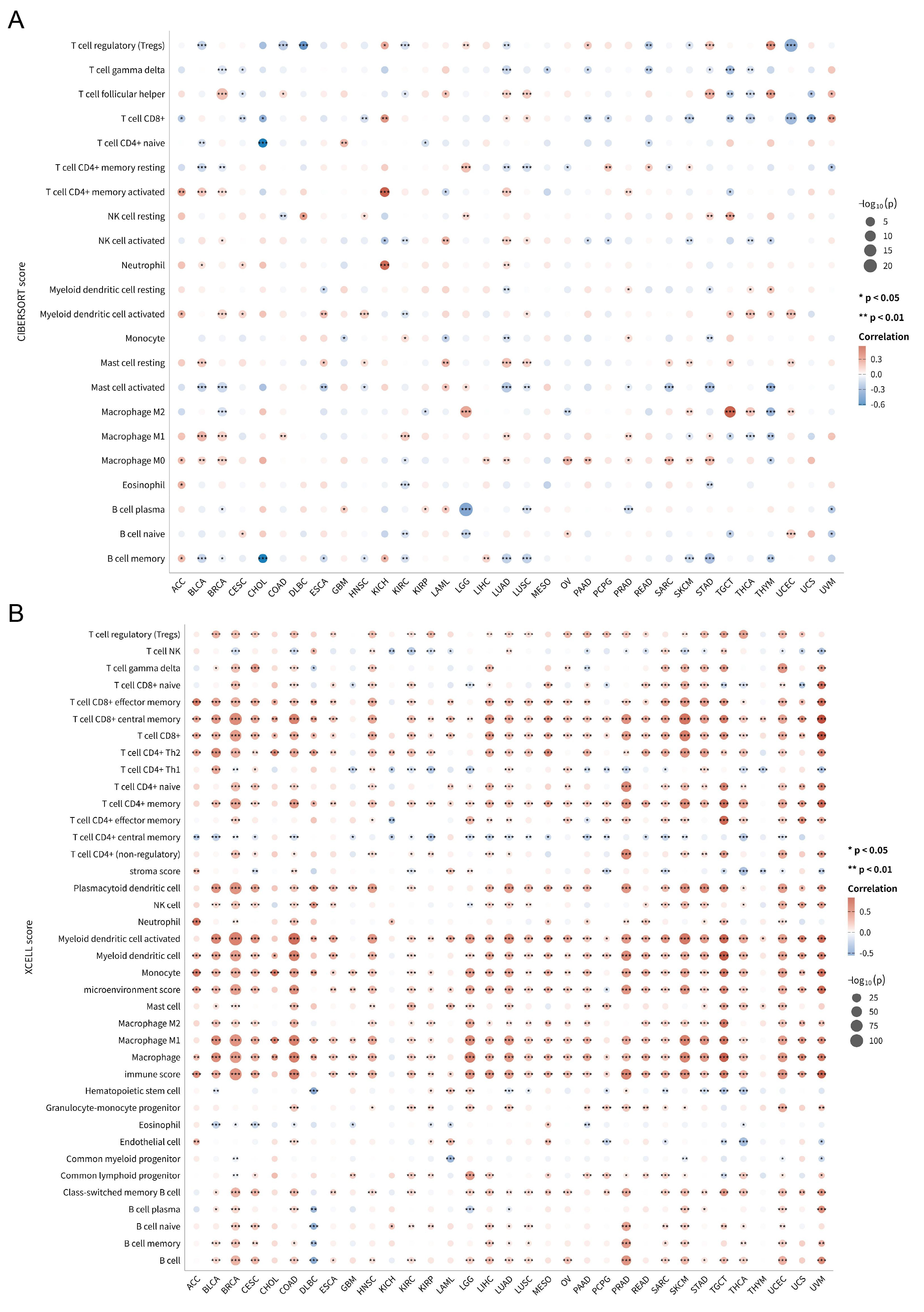
**

**Figure S4 Association of HDGF with tumor immune infiltration.** (A-B) Heatmap generated from the TIMER database showing the correlation between HDGF expression and the abundance of six immune cell types across 30 cancer types. * *P* <0.05, ** *P* <0.01, *** *P* <0.001.

**
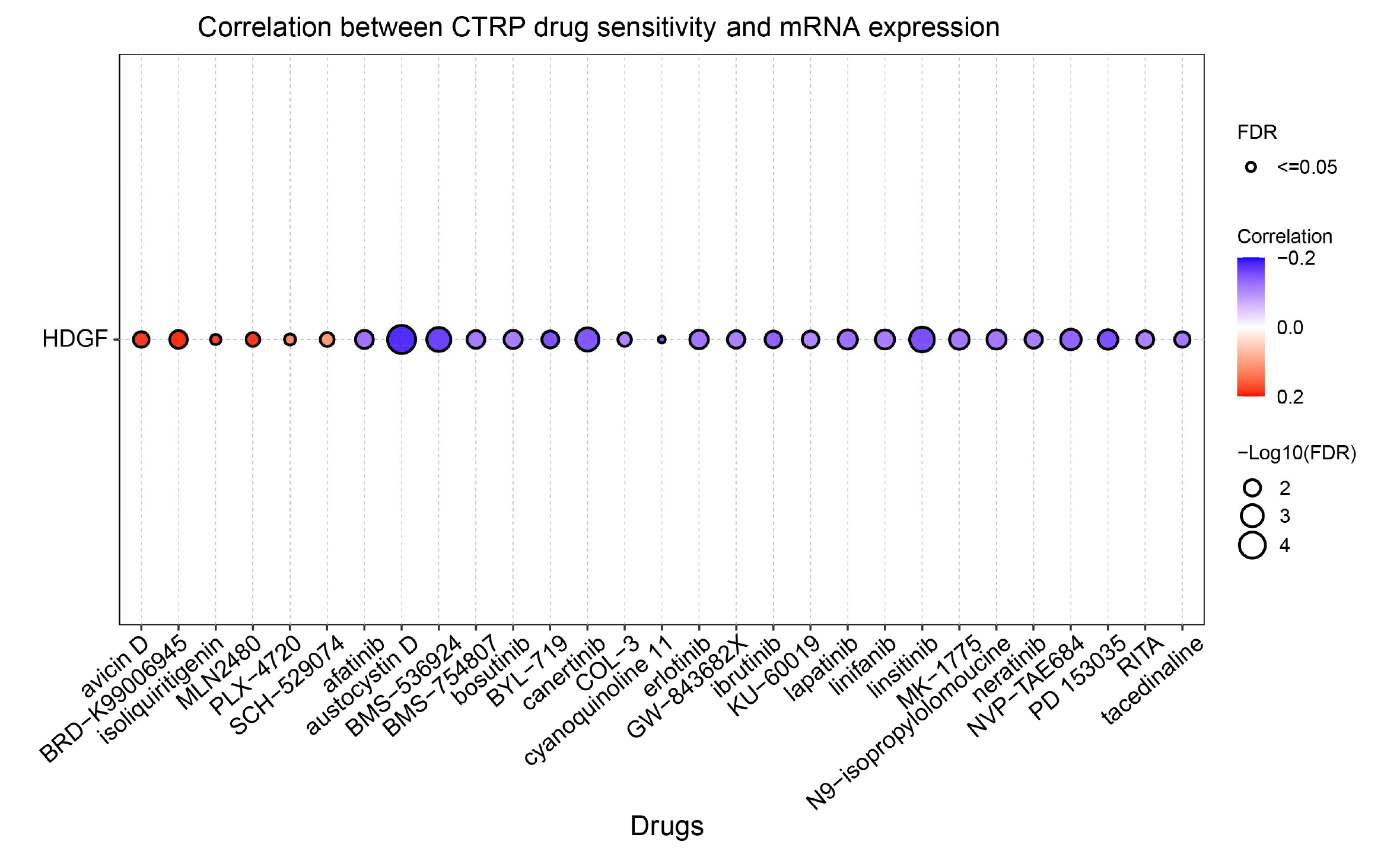
**

**Figure S5 Drug sensitivity analysis of HDGF.**
