# Supplementary figures and images for: Lactylation-driven metabolic reprogramming promotes osteosarcoma malignancy via HDGF-mediated proliferation and immune modulation
Source: Front Immunol. 2026 May 20;17:1836254. doi: 10.3389/fimmu.2026.1836254 (PMC13229866; doi:10.3389/fimmu.2026.1836254)

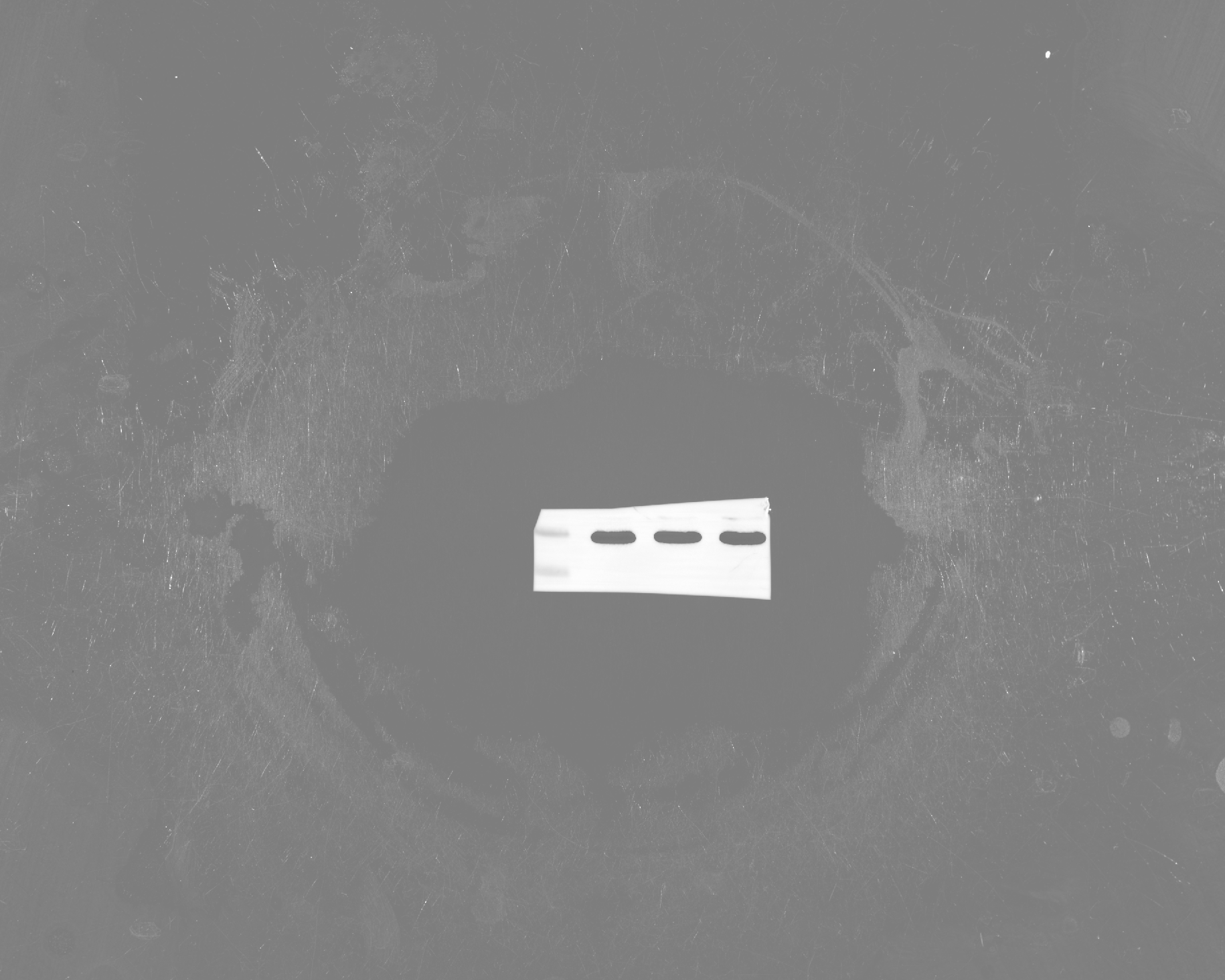

Supplement: Supplementary file 2 [file DataSheet2.zip › WB原图/143B GAPDH.tif]

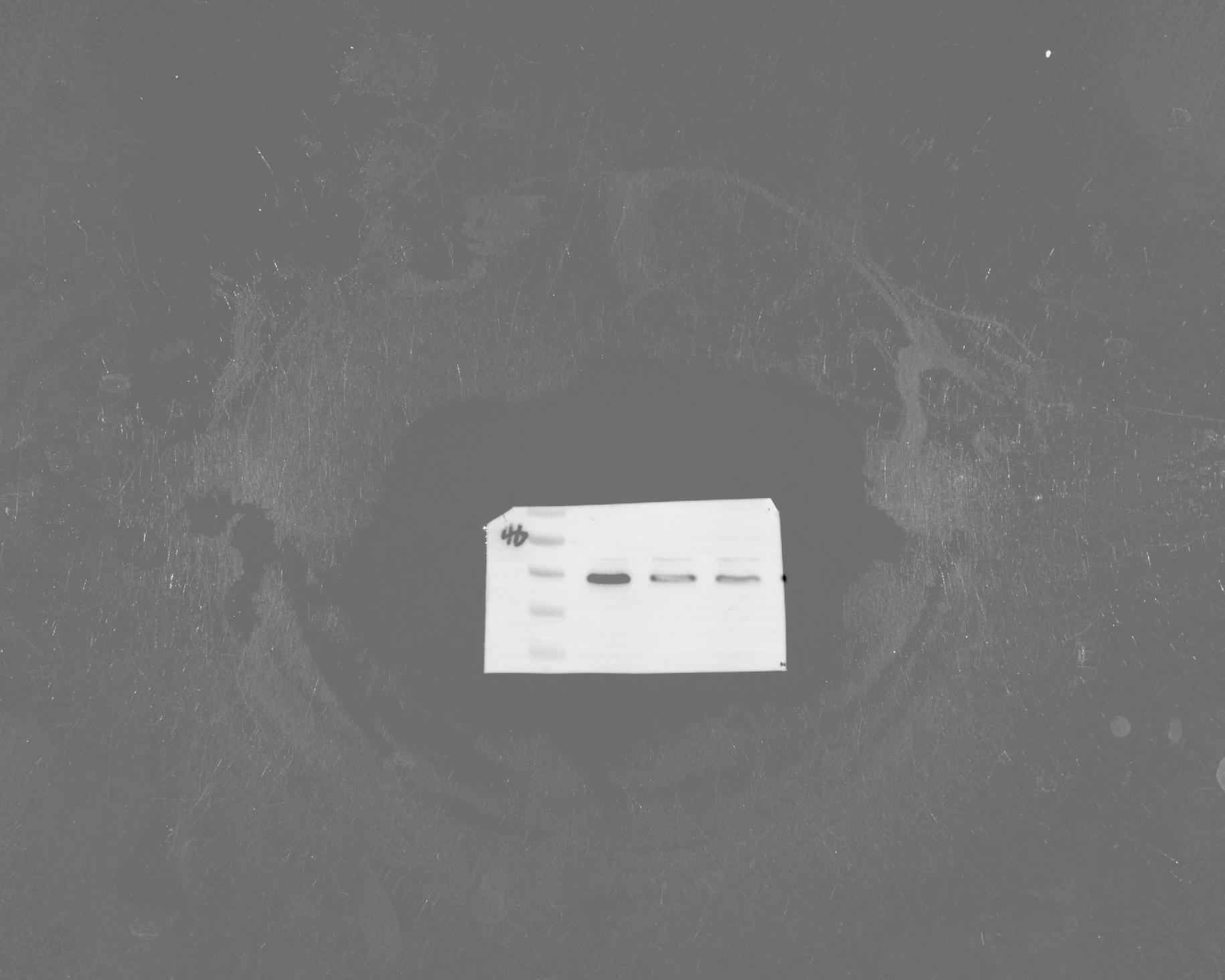

Supplement: Supplementary file 2 [file DataSheet2.zip › WB原图/143B HDGF.tif]

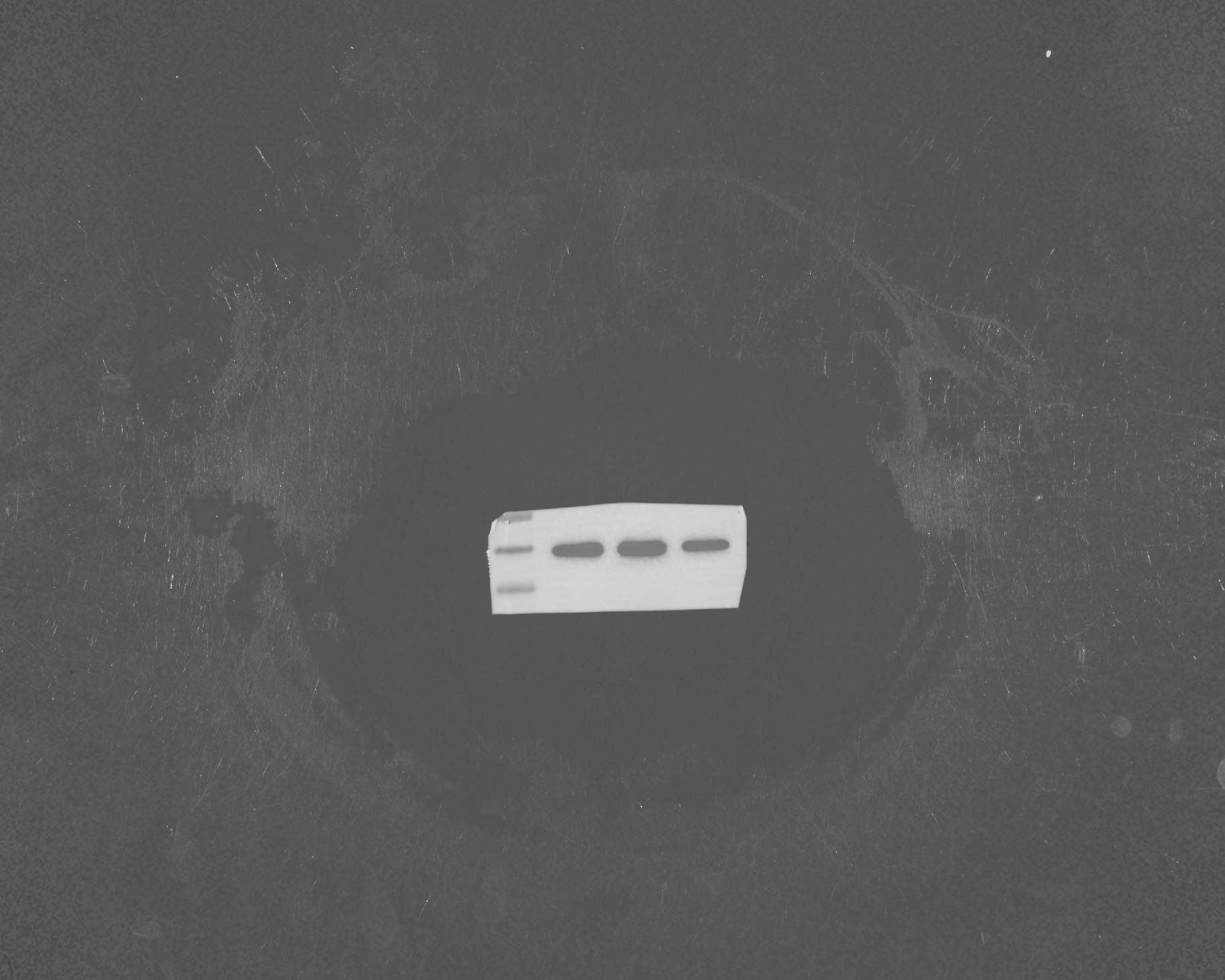

Supplement: Supplementary file 2 [file DataSheet2.zip › WB原图/U2OS GAPDH.tif]

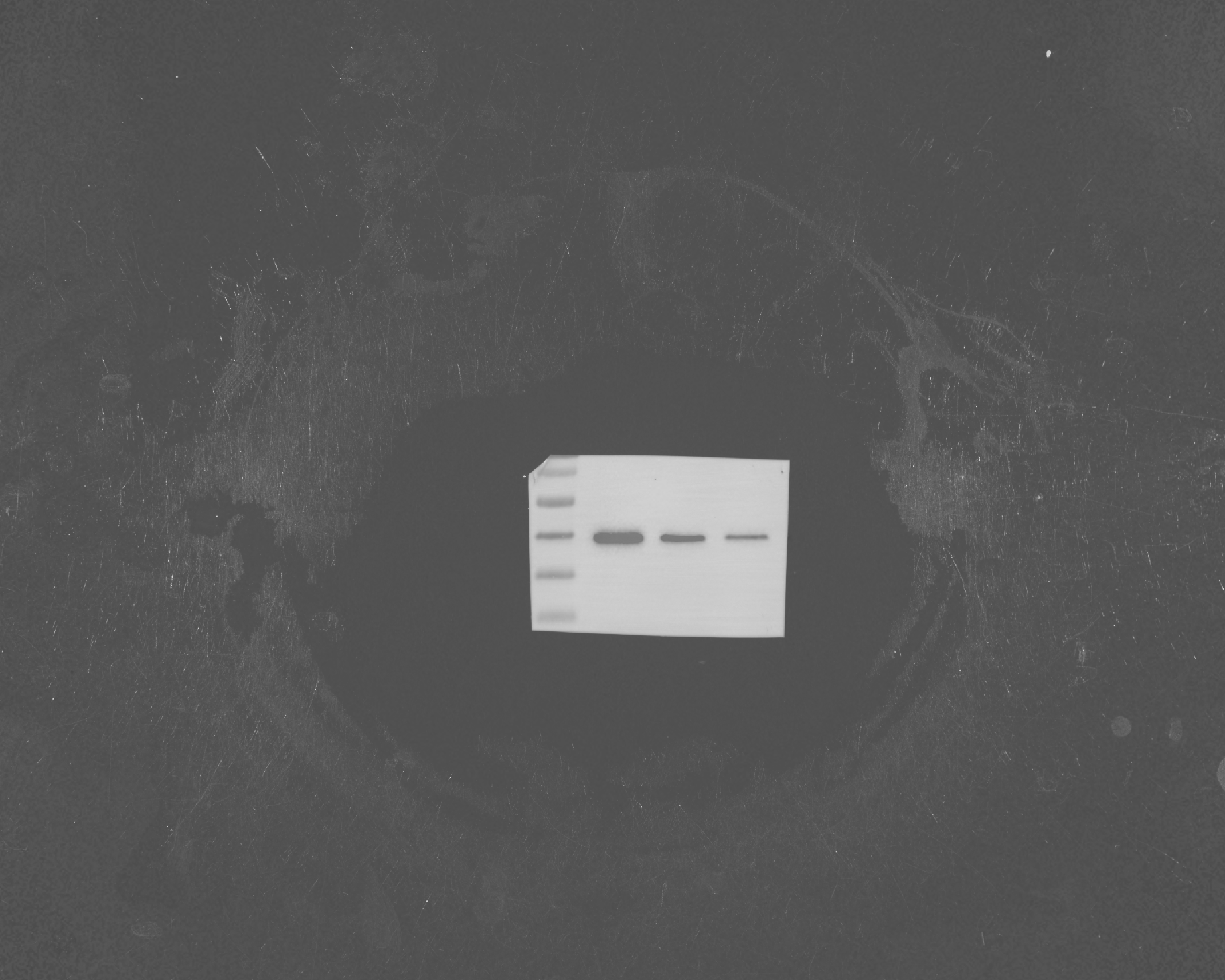

Supplement: Supplementary file 2 [file DataSheet2.zip › WB原图/U2OS HDGF.tif]
